# Results of a “GWAS Plus:” General Cognitive Ability Is Substantially Heritable and Massively Polygenic

**DOI:** 10.1371/journal.pone.0112390

**Published:** 2014-11-10

**Authors:** Robert M. Kirkpatrick, Matt McGue, William G. Iacono, Michael B. Miller, Saonli Basu

**Affiliations:** 1 University of Minnesota, Department of Psychology, Minneapolis, Minnesota, United States of America; 2 University of Minnesota, School of Public Health, Division of Biostatistics, Minneapolis, Minnesota, United States of America; NIH - National Institute of Environmental Health Sciences, United States of America

## Abstract

We carried out a genome-wide association study (GWAS) for general cognitive ability (GCA) plus three other analyses of GWAS data that aggregate the effects of multiple single-nucleotide polymorphisms (SNPs) in various ways. Our multigenerational sample comprised 7,100 Caucasian participants, drawn from two longitudinal family studies, who had been assessed with an age-appropriate IQ test and had provided DNA samples passing quality screens. We conducted the GWAS across ∼2.5 million SNPs (both typed and imputed), using a generalized least-squares method appropriate for the different family structures present in our sample, and subsequently conducted gene-based association tests. We also conducted polygenic prediction analyses under five-fold cross-validation, using two different schemes of weighting SNPs. Using parametric bootstrapping, we assessed the performance of this prediction procedure under the null. Finally, we estimated the proportion of variance attributable to all genotyped SNPs as random effects with software *GCTA*. The study is limited chiefly by its power to detect realistic single-SNP or single-gene effects, none of which reached genome-wide significance, though some genomic inflation was evident from the GWAS. Unit SNP weights performed about as well as least-squares regression weights under cross-validation, but the performance of both increased as more SNPs were included in calculating the polygenic score. Estimates from *GCTA* were 35% of phenotypic variance at the recommended biological-relatedness ceiling. Taken together, our results concur with other recent studies: they support a substantial heritability of GCA, arising from a very large number of causal SNPs, each of very small effect. We place our study in the context of the literature–both contemporary and historical–and provide accessible explication of our statistical methods.

## Introduction

### Candidate-Gene Association

General cognitive ability (GCA) is that mental capacity which is involved to some extent in every cognitively demanding task. It is an important individual-differences variable, and correlates non-trivially with other variables in a considerable variety of domains [1], [2], [3], [4]. Decades of research from twin, family, and adoption studies have established that general cognitive ability (GCA) is a substantially heritable trait. Estimates of its heritability (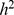), the proportion of its variance that is attributable to genetic factors, typically range from 0.50 to 0.70 [5], [6], [7], and are sometimes as high as ∼0.80 [8]. In light of the empirical fact that genes influence cognitive ability, a natural subsequent question to ask is *which* genetic polymorphisms contribute to individual variation in the trait

Association analysis is merely a test for whether the allelic state of a genetic polymorphism systematically covaries with the disease or quantitative trait of interest (typically via regression analysis). It can implicate a specific polymorphism provided that the “causal” polymorphism actually be typed, or alternately, lie in close chromosomal proximity–linkage disequilibrium (LD)–to a marker that is typed. (Linkage disequilibrium is the logical consequence of recombination, over many generations, in entire populations. The result is that loci very close to one another on a chromosome are least likely to be sundered by a recombination event, and therefore, polymorphisms within small “blocks” of DNA on a given chromosome tend to be transmitted together in the population. This essentially induces correlation between markers in tight proximity to one another on the same chromosome.) For a number of years, the dense genotyping needed for association analysis was costly, so association analysis saw use primarily in candidate-gene studies.

The rationale behind the candidate-gene study is simple: typing markers within genes that are *a priori* plausibly related to the phenotype is a focused use of limited genotyping resources, which is (presumably) more likely to identify genetic variants that are truly associated with the phenotype. Unfortunately, the candidate-gene association literature has been plagued by apparent false positives that fail to replicate. This has occurred in human genetics at large [9], [10], and has occurred in candidate-gene association research for GCA since its inception [11]. In fact, one recent article concluded that “most reported genetic associations with general intelligence are probably false positives”[12].

Rather presciently, Risch & Merikangas [13] foreshadowed the advent of the genome-wide association (GWAS) study in their remark that an “approach (association studies) that utilizes candidate genes has far greater power, *even if one has to test every gene in the genome*” (p. 1516, emphasis supplied). The genome-wide association scan (GWAS) grew naturally out of researchers’ (1) demand for denser and denser coverage of variation in more and more genes, and (2) growing dissatisfaction with replication failures in association studies of *a priori* biologically-hypothesized candidate genes. GWAS in the modern sense involves typing individuals on at least 300,000 SNPs throughout the genome [14]; due to LD, SNPs that are typed can “speak on behalf” of non-genotyped SNPs and other polymorphisms that are nearby on the same chromosome. It is only within the past five years or so that biotechnology reached such sophistication that researchers can feasibly genotype a sample of participants on hundreds of thousands of SNPs, and engage in the atheoretical brute-force empiricism that is GWAS. Needless to say, there is an inherent multiple-testing problem in GWAS; the currently accepted standard for “genome-wide significance” is *p*<5×10^−8^.

### GWAS

In a sense, the IQ QTL Project [15] carried out the first “genome-wide association study” of GCA (via DNA pooling [16]), with only 1,847 markers; it failed to uncover replicable association. A “low-density GWAS” for IQ has been reported by Pan, Wang, & Aragam [17], with no genome-wide significant hits. The first two “true” GWAS for GCA both used samples of children from the Twins Early Development Study (TEDS). Butcher, Davis, Craig, and Plomin [18] reported the first; subsequently, Davis et al. [19] ran a similar study. Butcher et al. reported that, at the uncorrected 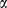 = 0.05, their full-sample association analysis would have 100%, 98%, and 71% power to detect an additive SNP accounting for 1%, 0.5%, and 0.2%, respectively, of the phenotypic variance. With a Bonferroni correction for 28 hypothesis tests yielding a per-comparison 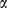 = 0.001786, Davis et al.’s full-sample association analysis would have 99.5% and 82% power to detect a SNP accounting for 1% and 0.5%, respectively, of the phenotypic variance.

Butcher et al. [18] observed nominally significant association from 6 of 37 SNPs entered into the full-sample association analysis. After implementing Benjamini and Hochberg’s [20] step-up procedure to control false discovery rate, only one of these SNPs, rs1378810, was resolved as a discovery (*r* ^2^ = 0.004, corrected *p*<0.03). Of Davis et al.’s [19] 28 SNPs entered into the full-sample association analysis, 9 were nominally significant, but none survived Bonferroni correction or Benjamini and Hochberg’s procedure.

The largest effect-size estimate that Davis et al. [19] reported is *r* ^2^ = 0.0024. The largest effect-size estimate that Butcher et al. [18] reported is *r* ^2^ = 0.004; the sum of effect sizes of their six nominally significant SNPs was only 1.2% of the variance. Butcher et al commented accordingly, and succinctly summarized the main lesson of GWAS for quantitative traits (p. 442, emphasis in original):

One possible reason for not observing larger, common, single-locus SNP effects for *g* is that they do not exist…[I]t may be that for…quantitative traits…the main finding is the *exclusion* of SNPs of large effect size to the extent that coverage for common variants is virtually complete…[W]innowing the wheat from the chaff will be difficult, requiring extremely large samples, multiple-stage designs, and replication in independent samples.

As others have pointed out, the same lesson is apparent from GWAS for human height [21], [22]. Height is highly heritable, uncontroversial in definition, and easily measured, almost without error. And yet, the SNPs identified by initial GWAS for height (reviewed in Ref [22]) each accounted for around 0.3% or less of the phenotypic variance, and in total, 3%. It would appear that variation in quantitative traits is attributable to a very large number of polymorphisms of very small effect. (Non-additivity of genetic effects is another possible explanation. However, this appears to be unlikely for GCA, since there is little evidence of non-additive genetic variance from twin, family, and adoption studies of this trait [23]).

Clearly, it is necessary to move beyond analyses of one SNP at a time. We refer to GWAS, combined with analyses that aggregate across multiple SNPs in some fashion, as “GWAS plus.” We describe three such multi-SNP analyses: *VEGAS*, polygenic scoring, and *GCTA*.

### GWAS Plus: Polygenic Scoring

Both TEDS GWAS [18], [19] illustrated a simple approach to combining the effect of multiple SNPs: for each participant, aggregate those alleles suggestively implicated in the GWAS into a “genetic score” for him/her. From the six nominally significant SNPs from the GWAS, Butcher et al. simply counted how many of the putative increaser alleles each participant carried. This score ranged from 1 to 11 in the subsample of 2,676 children in which it was calculated, and correlated *r* ≈0.10 with general ability–a very significant result (*p*<3×10^−8^). Similarly, Davis et al. created a score from the nine nominally significant SNPs from the GWAS, which ranged from 6 to 16, and accounted for 1.2% of phenotypic variance. Davis et al. acknowledge that they conducted the genetic scoring analysis with the same participants in which they conducted the GWAS, so the analysis is almost certainly capitalizing on chance.

S. Purcell (with the International Schizophrenia Consortium [24]) appears to be the first to have performed genetic scoring by weighting each selected SNP by its GWAS regression coefficient, and cross-validating in a separate sample. Not surprisingly, the genetic score’s predictive performance upon cross-validation depended upon the GWAS *p*-value threshold set for a SNP to be included toward the score (Ref [24], supplemental online material); at best, the genetic score could predict around 3% of disease risk in the cross-validation sample.

Lango Allen et al. [25] (the GIANT Consortium) utilized genetic scoring subsequent to a GWAS for human height on a titanic scale: a combined sample of 133,653 participants, with called or imputed genotypes on over 2.8 million SNPs, and a replication sample of 50,074 participants. The GIANT Consortium ultimately identified 180 SNPs robustly associated with height. The genetic score from these loci predicted around 10% of the phenotypic variance in each cross-validation sample. When additional SNPs at varying significance thresholds were counted toward the score, it predicted as much as 16.8% of the variance in a cross-validation sample.

### GWAS plus: *VEGAS*

*VEGAS* (Versatile Gene-based Association Study [26]) is a program that tests each *gene* (specifically, all genotyped SNPs in each gene) for association with the phenotype, via parametric bootstrapping. A rather clever program, it takes GWAS results as its input, requiring only the rs numbers and GWAS *p*-values of each SNP. If an Internet connection is available, the program “knows” (from bioinformatic databases) which of 17,787 autosomal gene(s), if any, contain each SNP. Within each gene, the program first converts each SNP *p*-value to the corresponding quantile from a central chi-square distribution on 1*df*, and sums them to produce an observed test statistic *T_obs_* for that gene. The null hypothesis is that there is no association of any SNP in the gene with the phenotype. Under the null, and if there were zero LD among the gene’s *m* SNPs, then 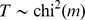. Under the null, but at the other extreme of perfect LD among the *m* SNPs, then 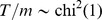.

But, *VEGAS* also “knows” the LD structure from reference datasets for three populations: HapMap CEU (Caucasians of European ancestry), CHB and JPT (Han Chinese and Japanese), and YRI (West Africans). *VEGAS* assumes that, under the null hypothesis, the LD pattern among SNPs in a gene dictates the correlation pattern among the single-SNP test statistics–an assumption made, for example, in methods for controlling Type I Error rate in association studies of SNPs in LD with one another [27], [28]. The matrix of pairwise LD correlations for the user-specified population, **Σ**, is employed in the random generation of test statistics under the null hypothesis. Specifically, in each iteration, an order-*m* vector is drawn from a multivariate normal distribution with zero mean and covariance matrix equal to **Σ**. The elements of this vector are squared and summed, yielding the value of the test statistic for that iteration. The proportion of test statistics exceeding *T_obs_* provides the *p*-value for the gene-based test of association. Liu et al. [26] recommend a Bonferroni-corrected significance level of *p*<0.05/17,787, or 2.8×10^−6^, which is slightly conservative since genes’ boundaries overlap to some extent.

### GWAS Plus: *GCTA*

*GCTA* (Genome-wide Complex Trait Analysis) [29] is a software package that implements what some [30] have referred to as GREML, for “genomic-relatedness restricted maximum-likelihood.” Instead of regressing a quantitative trait onto one marker at a time, *GCTA* instead assesses how much of the phenotypic variance is attributable to *all* the typed markers at once, which is accomplished by treating all the markers as random effects, and entering them into a mixed linear model fit by restricted maximum likelihood. *GCTA* thereby provides an unbiased estimate of the variance attributable to the typed SNPs, and a matrix of (roughly) genome-wide SNP correlations among participants–a genetic relationship matrix, obtainable from a genotyped sample of classically unrelated participants. Put simply, *GCTA* attempts to predict phenotypic similarity among individuals from their genetic similarity, and to predict phenotypic variance that would otherwise be treated as error. *GCTA* may be expected to outperform polygenic scoring, because it does not rely upon estimates of individual SNP effects, which are prone to sampling error [31].

For *n* participants typed on *m* SNPs, the *GCTA* model [29] is expressed as
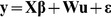
(1)where **y** is a random *n*×1 vector of scores on a quantitative trait, **X** is a model matrix of scores on covariates, **β** is a vector of the covariates’ regression coefficients (fixed effects), and residual vector 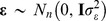. Further, **u** is an *m*×1 vector of random SNP effects, such that 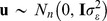; **W** is an *n*×*m* matrix of participants’ reference-allele counts, expressed as *z*-scores (i.e., columns are standardized).

We hereby condition upon the observed value of **X**. Since the random effects have zero expectation, 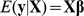. Now define the phenotypic variance matrix, **V**:
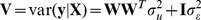
(2)


Matrix **V** is the model-predicted covariance matrix of participants’ phenotype scores. Intuitively, each off-diagonal (covariance) element of **V** is the degree of phenotypic similarity between the two participants, as predicted from their genotypic similarity. Now, further define genetic relationship matrix, **A**:
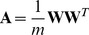
(3)


Matrix **A** is *n*×*n*, and roughly, may be regarded as a matrix of correlations between different participants’ genotypes. However, this is not strictly correct, since **W** is standardized by column (SNP) rather than by row (participant), and therefore the elements of **A** may exceed unity in absolute value.

Let 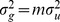, the variance attributable to all SNPs. The model may now be written:
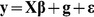
(4)where **g** is an *n*×1 vector of random genetic effects, distributed as 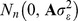. Now,
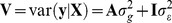
(5)Where 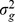 is the component of variance attributable to all typed SNPs and all untyped “causal” mutations in close LD with them. The model-predicted phenotypic variance 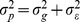. Herein, we refer to the ratio of 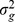 to 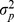 as 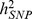, for it is a lower bound on the additive heritability of the phenotype. Estimation is carried out via restricted maximum-likelihood; details of the algorithm may be found in Ref [29].

### Recent Developments

Davies et al. [32] reported a “GWAS Plus” for cognitive abilities. The discovery sample contained 3,511 unrelated participants, combined from 5 cohorts of older adults in the United Kingdom. The replication cohort comprised 670 Norwegian participants of a wide range of ages (18–79). Davies et al. extracted composite scores for both crystallized and fluid ability from the ability measures in each cohort, and conducted separate analyses for fluid and crystallized ability.

Davies et al. [32] combined association results from the 5 UK cohorts via meta-analytic techniques. No single SNP achieved genome-wide significance (*p*<5×10^−8^). Gene-based tests in *VEGAS* implicated only one gene, *FNBP1L*, which was not confirmed in the replication cohort. Davies et al. performed polygenic scoring using the most lenient SNP inclusion threshold possible: *all* genotyped SNPs, irrespective of GWAS *p*-value. In the UK samples, this score predicted between 0.45% and 2.19% of the variance. Under cross-validation in the replication cohort, this score predicted less than 1% of the variance (statistically significant for both fluid and crystallized ability). Davies et al. emphasized that, when treating SNPs as single fixed effects, their individual effect sizes will be quite small, and estimated with considerable sampling error.

Instead, *GCTA*, though it is silent with regard to the individual contribution of each marker, treats all SNPs as random effects and estimates a single omnibus variance component. (In this report, we are chiefly interested in the simplest application of *GCTA*, when only variance components and fixed-effects regression coefficients are computed. If the original genotypic data used to calculate **A** is available, then it is also possible to obtain empirical best linear unbiased predictions (eBLUPs) of the individual SNP effects in vector **u** from Equation (1).) This seems to be one of its major advantages. In any event, the most notable results from Davies et al. [32] were from *GCTA*, which produced variance-component estimates equivalent to 40% of the variance in crystallized ability, and 51% of the variance in fluid ability. Davies et al. (p. 1) conclude that “human intelligence is highly heritable and polygenic.”.

A recent study of GCA in children and adolescents reported by the Childhood Intelligence Consortium (CHIC)[33] has borne out that same conclusion. The CHIC study represented a collaboration of six discovery cohorts (total *N* = 12,441) and three replication cohorts (*N* = 5,548). One of the replication cohorts was a sample of Caucasian adolescent participants from studies conducted at the Minnesota Center for Twin & Family Research (MCTFR, *N* = 3,367), which is a subset of the present study’s sample. The phenotype in all cohorts was either Full-Scale IQ score or a composite score derived from a battery of both verbal and non-verbal tests. GWAS SNP results were combined across discovery cohorts by meta-analysis. No SNP reached genome-wide significance. Among the top 100 SNPs from the discovery GWAS, none was significant after Bonferroni correction in any of the replication cohorts, though discovery sample’s estimated regression coefficients for these 100 SNPs were moderately positively correlated with those from two of the three replication cohorts, but not the MCTFR cohort.

Gene-based analysis with *VEGAS* in Benyamin et al.’s [33] discovery sample suggested association with *FNBP1L* (*formin binding protein 1-like*, on chromosome 1; *p* = 4×10^−5^), which “is involved in a pathway that links cell surface signals to the actin cytoskeleton” (p. 3). This was also the most significantly associated gene in Davies et al.’s [32] discovery cohort. However, one cohort was common to both studies–Davies et al. used adult IQ scores from the Lothian Birth Cohorts, whereas Benyamin et al. used their childhood IQ scores. When Benyamin et al. combined *VEGAS* results across all of their cohorts except the Lothian Birth Cohorts, the association with *FNBP1L* remained nominally significant (*p* = 0.0137), as did the top SNP in the gene (*p* = 4.5×10^−5^). Benyamin et al. regarded this as robust evidence of association between GCA and polymorphisms in *FNBP1L*.

Benyamin et al. [33] also reported results of polygenic scoring analyses conducted in the replication cohorts. These analyses calculated polygenic scores from the SNP regression weights obtained in the meta-analytic GWAS results from the discovery sample. Eight such analyses were conducted in each replication cohort, with a different *p*-value cutoff for each. That is, polygenic score for each such analysis was computed from a set of SNPs the *p*-values of which exceeded some threshold in the discovery sample. The proportion of variance attributable to the polygenic score varied by *p*-value cutoff and by replication cohort, but was statistically significant for at least one analysis in each replication cohort. The best achieved in the MCTFR cohort was 0.5% of variance (*p* = 5.52×10^−5^). Finally, Benyamin et al. reported *GCTA* results for the three largest cohorts in the study, one of which was the MCTFR cohort. Estimates of 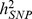 varied from 0.22 to 0.46, with the MCTFR estimate in between at 0.40; all three estimates were significantly different from zero. Based on all results, Benyamin et al. conclude that “[c] hildhood intelligence is heritable, highly polygenic and associated with *FNBP1L*” (p. 1).

In the present study, we report the detailed results of our “GWAS Plus” from our *full* sample of 7,100 Caucasian MCTFR participants, both adolescents and adults. We conducted our GWAS using over 2.6 million SNPs and a method appropriate for the complicated family structures in our dataset. We then conducted gene-based association tests in *VEGAS* with the SNP *p*-values calculated in our GWAS. We also carried out polygenic scoring analyses with five-fold cross-validation, at different *p*-value cutoffs (*a la* Benyamin et al. [33]) and under two different schemes of weighting SNPs to compute the score. Finally, we ran *GCTA* to estimate how much of the phenotypic variance is attributable to all genotyped SNPs.

## Methods

### Ethics Statement

Both longitudinal family studies, the Minnesota Twin Family Study (MTFS) and the Sibling Interaction & Behavior Study (SIBS), and the collection, genotyping, and analysis of DNA samples, were approved by the University of Minnesota Institutional Review Board's Human Subjects Committee. Written informed assent or consent was obtained from all participants; parents provided written consent for their minor children.

### Sample

#### Participants

Our participants came from two longitudinal family studies conducted by the MCTFR. MTFS [34], [35], [36] is a longitudinal study of same-sex twins, born in the State of Minnesota between 1972 and 1994, and their parents. There are two age cohorts in this community-based sample, an 11-year-old cohort (10–13 years old at intake, mean age = 11.78) and a 17-year-old cohort (16–18 years old at intake, mean age = 17.48). Zygosity has been genomically confirmed for all DZ twins included in the present study [37]. SIBS [38] is a longitudinal adoption study of sibling pairs and their parents. This community-based sample includes families where both siblings are adopted, where both are biologically related to the parents, or where one is adopted and one is biologically related. As required by SIBS inclusion criteria, any sibling in the sample who was adopted into the family will not be biologically related to his or her co-sibling, which has been genomically verified for all SIBS participants in the present study [37]. The age range at intake was 10–19 for the younger sibling, and 12–20 for the older. For the purposes of our analyses, the sample comprises six distinct family types:

1. Monozygotic- (MZ) twin families (*N* = 3,939 in 1143 families),
2. Digyzotic- (DZ) twin families (*N* = 2,114, in 638 families),
3. SIBS families with two adopted offspring (*N* = 291, in 224 families),
4. SIBS families with two biological offspring (*N* = 472, in 184 families),
5. “Mixed” SIBS families with 1 biological and 1 adopted offspring (*N* = 204, in 107 families),
6. Step-parents (*N* = 80).

As explained below, our method of analysis accounted for the clustering of individual participants within families. However, family-type #6, step-parents, do not fit neatly into a four-member family unit; we treated them as independent observations (in a sense, as one-person families) in our analysis. A total of *N* = 7,100 participants were included in our analyses. Descriptive characteristics of the sample are provided in Table 1. Details concerning families’ patterns of data availability are provided in Table S1. Genotype and phenotype data have been submitted to dbGaP (accession number phs000620.v1.p1).

**Table 1:** Descriptive characteristics of Study #1 sample.

	Parents	Twins (17yo)	Twins (11yo)	Non-twin Biological Offspring	Adoptees	Step-parents
*N*	3,264	1,146	2,080	414	116	80
Female(%)	60.2%	55.3%	50.1%	52.2%	46.6%	8.8%
Mean Age at Intake (SD)	43.3 (5.46)	17.5 (0.45)	11.8 (0.43)	14.9 (1.89)	15.1 (2.17)	40.6 (7.45)
Mean FSIQ (SD)	105.8 (14.2)	100.4 (14.1)	103.6 (13.5)	108.5 (13.1)	105.7 (14.3)	103.4 (15.7)

Table notes: Total *N* = 7100, in 2376 families. FSIQ = Full-Scale IQ; 17yo = 17-year-old cohort; 11yo = 11-year-old cohort. For a minority of twins (38%), FSIQ represents a within-person average of FSIQ scores from more than one assessment (see text). FSIQ range: 151–59 = 92. Parental intake age range: 65–28 = 37. Offspring intake age range: 20–10 = 10.

#### Genotyping

Participants who provided DNA samples were typed on a genome-wide set of markers with the Illumina Human660W-Quad array. Both DNA samples and markers were subject to thorough quality-control screens. 527,829 SNPs on the array were successfully called and passed all QC filters, which filters include call rate <99%, minor allele frequency <1%, and Hardy-Weinberg equilibrium *p*-value<10^−7^. After excluding DNA samples that failed quality-control screening, a genotyped GWAS sample of 8,405 participants was identified.

Population stratification occurs when one’s sample of participants represents heterogeneous populations across which allele frequencies differ appreciably, and can produce spurious genetic association (or suppress genuine association). We therefore restricted our analyses only to participants who are Caucasian, of European ancestry (“White”), based upon both self-reported ancestry as well as principal components from EIGENSTRAT [41]. These principal components were extracted from an *n*×*n* covariance matrix of individuals’ genotypes across SNPs (similar to matrix **A** described above). A White GWAS sample of 7,702 participants was identified. The sample for the present study is the 7,100 out of 7,702 White participants with available phenotype data. Details concerning genotyping, quality-control, and ancestry determination can be found in Ref [37].

#### Imputation

Many known SNPs exist that are not on our Illumina array. But, by combining observed SNP genotypes with what is known–*a priori,* from reference data–about haplotype frequencies in the population, the allelic state of common untyped SNPs can often be imputed with a high degree of accuracy. For SNP imputation, using HapMap2 [42] as the reference panel, we first phased our observed genotypes into expected haplotypes with *BEAGLE* [43], which takes information from genotyped relatives into account to improve phasing. We then input phased data into *Minimac*, a version of *MaCH* [44], to impute SNP states for a total of 2,094,911 SNPs not on the Illumina array. In our GWAS, we used the allelic dosages of these SNPs, which are individuals’ posterior expected reference-allele counts on each SNP so imputed. The quality of the imputation for an untyped SNP may be assessed by its imputation *R* ^2^ [44], which is the ratio of the variance of its imputed dosages to its population variance (from reference data). Our GWAS only included dosages of imputed SNPs with imputation *R* ^2^ >0.5, of which there were 2,018,818. Between these imputed SNPs and the 527,829 from the array, we analyzed a total of 2,546,647 SNPs in our GWAS.

#### Phenotypic measurement

Measurement of GCA was included in the design of the intake assessment for most participants, by way of an abbreviated form of the Wechsler Intelligence Scale for Children-Revised (WISC-R) or Wechsler Adult Intelligence Scale-Revised (WAIS-R), as age-appropriate (that is, 16 or younger, and older than 16, respectively). The short forms consisted of two Performance subtests (Block Design and Picture Arrangement) and two Verbal subtests (Information and Vocabulary), the scaled scores on which were prorated to determine Full-Scale IQ (FSIQ). FSIQ estimates from this short form have been shown to correlate 0.94 with FSIQ from the complete test [45]. Parents in the SIBS sample were an exception, in that they were not tested with this short form of WAIS-R until the first SIBS follow-up assessment. By design, only one parent per SIBS family returned for this follow-up, which was usually the mother. As a result, IQ data for SIBS fathers is very limited in its availability.

IQ-testing was also included in the design of the second follow-up for both age cohorts of MTFS twins, and for the fourth follow-up for the 11-year-old cohort. At these assessments, twins received a further abbreviated form of WAIS-R, consisting only of the Vocabulary and Block Design subtests, the scaled scores on which were again prorated to determine FSIQ. Of the 3,226 twins entered into our analysis, 903 were tested twice, and 337 were tested three times. Multiple testing occasions were spaced approximately seven years apart. To achieve a more reliable assessment of the phenotype, we simply averaged all available measures of FSIQ for each participant, and used these single within-person averages in analysis. FSIQ among participants entered into analysis ranged from 59 to 151 (also see Table 1). Twelve participants with FSIQ of 70 or below were included in analyses. Despite their low scores, these participants were not noticeably impaired and were capable of completing the multifaceted MTFS/SIBS assessment during their visit. They are therefore unlikely to meet diagnostic criteria for mental retardation [46], and instead, likely represent the low end of the normal-range distribution of GCA. (Participants who are discovered to have a physical or mental disability severe enough to prevent them from completing the intake assessment are retroactively ruled ineligible to participate. This has occurred for five MTFS twin pairs and one SIBS adoptee, whose data were eliminated from the studies’ databases.).

### Analyses

#### Statistical power

Because our participants are clustered within 2,376 families, our effective sample size is less than 7,100 participants. We conducted two sets of power calculations in *Quanto* [47], one that assumed 7,000 independent participants (an aggressive estimate of our effective sample size) and one that assumed 2,000 independent participants (a conservative estimate of our sample size). Both assume a Type I error rate of 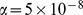, i.e. genome-wide significance. With 7,000 independent participants, our GWAS would have at least 80% power to detect a SNP accounting for 0.6% of phenotypic variance. With 2,000 independent participants, our GWAS would have at least 80% power to detect a SNP accounting for 2% of phenotypic variance

#### GWAS

Our GWAS consisted of a large number of least-squares regressions of FSIQ onto the genotype (or imputed dosage) of each SNP, along with covariates, which were sex, birth year, and the first 10 principal components from EIGENSTRAT [41], to control for any crypto-stratification (i.e., lurking population stratification in a sample of apparently homogeneous ancestry) within this White sample. (There are three reasons why we covaried out birth year, rather than age-at-testing. First, IQ tests are age-normed in the first place. Second, a minority of our twins would in a sense have more than one age-at-testing, since their FSIQ scores entered into analysis are actually within-person averages from more than one testing occasion. Third, the nuisance confound of chief concern is the Flynn Effect (first reported in Refs [39] and [40])–the secular trend of increasing IQ scores with each generation–which is directly related to birth year, and not to age *per se.* Surprisingly, at a glance, our data are not consistent with the Flynn Effect. In the covariates-only FGLS regression, the estimated coefficient for birth year was −0.09 (Table S2), indicating that later birth year corresponded on average to lower IQ.) One notable example of this kind of stratification came from a study [48] in which a SNP in the gene for lactase (*LCT*) was significantly, but spuriously, associated with height among European-Americans. Allele frequency for the SNP in question is known to vary among regions of Europe, and no association was observed when participants were matched on grandparental country-of-origin. Instead, the SNP appeared to mark participants’ ancestral origins along a northwest-southeast axis running through the continent of Europe.

Because our participants are clustered within families, they were not sampled independently. To further complicate matters, the within-family covariance structure will depend upon the kind of family in question. We therefore employed a feasible generalized least-squares (FGLS) method in our GWAS, via *RFGLS*, a package for the R statistical computing environment designed for FGLS regression in datasets with complicated family structures [49]. (As is widely known (see Ref [49]), in multiple regression, when the residuals are uncorrelated and have mean zero and constant variance, the best linear unbiased estimate of the regression parameters is obtained as 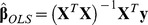; if the residuals are further normally distributed and stochastically independent, 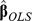 is also the maximum-likelihood estimator. If the residuals are not uncorrelated, 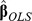 will not be maximally efficient, and the degrees-of-freedom for its test statistics will be mis-specified. In practice, the (non-diagonal) residual covariance matrix must be estimated from data. If **V** is a consistent such estimator, then the feasible generalized least-squares estimator of the regression coefficients is obtained as 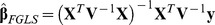).

*RFGLS* has a “rapid-FGLS” approximation, which we used to run the GWAS and which works as follows. First, an FGLS regression of the phenotype onto covariates only is run, in which the regression coefficients and the residual covariance matrix are both estimated. Then, that residual covariance matrix is saved to disk, so it can then be “plugged in” for use in all subsequent single-SNP regressions, with covariates. The approximation saves a considerable amount of computation time, since the residual covariance matrix is calculated only once. It produces negligible bias in the resulting *p*-values, so long as no SNP accounts for more than 1% of phenotypic variance [49] (which is a very reasonable assumption).

#### GWAS Plus: *VEGAS*

We conducted gene-based association tests in *VEGAS*, inputting 2,485,149 autosomal SNPs, both observed and imputed, and specifying HapMap CEU as the reference data for pairwise LD correlations. We also ran *VEGAS* inputting only the 515,385 autosomal SNPs on the Illumina array.

#### GWAS Plus: polygenic scoring

We conducted polygenic scoring with five-fold cross-validation. Since the family is the independent unit of observation in our dataset, we first randomly divided the sample into five subsamples of approximately equal numbers of families, and with each family type approximately equally represented in each. Then, we ran a GWAS with the observed SNPs only, five times over, each time including four of the five subsamples–the calibration sample for that iteration. Then, the left-out subsample served as that iteration’s validation sample.

Each iteration, we used *PLINK* [50] to produce polygenic scores for the participants in the validation sample based on the GWAS statistics from the calibration sample, at the same eight *p*-value cutoffs used by Benyamin et al. [33]: *p*≤0.001, *p*≤0.005, *p*≤0.01, *p*≤0.05, *p*≤0.1, *p*≤0.25, *p*≤0.5, and *p*≤1 (i.e., all SNPs). We used two different weighting methods to calculate polygenic scores. The first simply used the GWAS regression coefficients from the calibration sample. The second weighted each SNP as either −1 or 1, depending on the sign of its coefficient. Thus, with eight *p*-value cutoffs and two weighting schemes, we produced 16 polygenic-score vectors in each validation sample.

To evaluate the performance of the polygenic scores under cross-validation in each iteration, we first ran a FGLS regression of the phenotype onto covariates only in the validation sample, and retained the residualized FSIQs. We then did another FGLS regression of the residualized FSIQ onto polygenic score, from which we calculated Buse’s *R* ^2^ [51]. Buse’s *R* ^2^ is the coefficient of determination from OLS regression, except that each sum is instead replaced by a quadratic or bilinear form in the vector of terms, with a weight-matrix coefficient (for our purposes, this weight matrix is the inverse of the residual covariance matrix obtained from regressing the residualized phenotype onto the score). (We also calculated Nagelkerke’s [52] generalized *R* ^2^, and the squared Pearson correlation between polygenic score and residualized phenotype. Nagelkerke’s *R* ^2^ was typically very close to Buse’s. The squared Pearson correlation was generally close to the other two, but tended to be higher, sometimes as much as twice as high as Buse’s).

With parametric bootstrapping, we assessed the performance of polygenic scoring under the null hypothesis of residualized FSIQ being independent of the SNPs, as follows. First, new phenotype scores were simulated by generating a new residual vector for each family and adding it to the family’s vector of predicted scores from the GWAS covariates-only regression. Each new residual vector was drawn from a multivariate-normal distribution with zero mean and covariance matrix as estimated for that family in the covariates-only regression. Thus, in each newly simulated dataset, the within-family covariance structure and the associations among covariates and phenotype in the real data are expected to be preserved, but the phenotype is generated independently of SNP genotypes (conditional on covariates). However, the procedure does assume that multivariate normality is a reasonable distribution for the residuals.

The simulated sample was then randomly divided so that 80% of families were assigned to the calibration subsample, and the remaining 20% to the validation subsample. A GWAS was then run in the calibration sample, the results of which were used to conduct polygenic scoring in the validation sample, in the same way as done in the real data. We repeated this process a total of only 50 times, as it was somewhat computationally demanding.

#### GWAS Plus: *GCTA*

We first computed the genetic relationship matrix **A** from all 7,702 White participants with genome-wide SNP data (which includes those for whom FSIQ scores were not available), using the 515,385 autosomal SNPs passing QC. We then ran *GCTA* to estimate 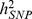, with FSIQ as the phenotype, and with the same covariates as used in the GWAS, as fixed effects. An exploratory analysis involving *GCTA* (described in Material S1) showed that including close relatives in the analysis can upwardly bias 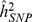 by confounding variance attributable to genotyped SNPs with variance attributable to shared environment. We therefore restricted the analysis only to participants whose degrees of genetic relatedness (from **A**) were 0.025 or smaller. To assess how well the distributional assumptions of the GREML method were met, we computed empirical best linear unbiased predictions (eBLUPs) of participants’ total genetic effects–**g** in Equation (4)–and residuals, both of which are assumed to be normally distributed.

## Results

### GWAS

**Figure 1:**
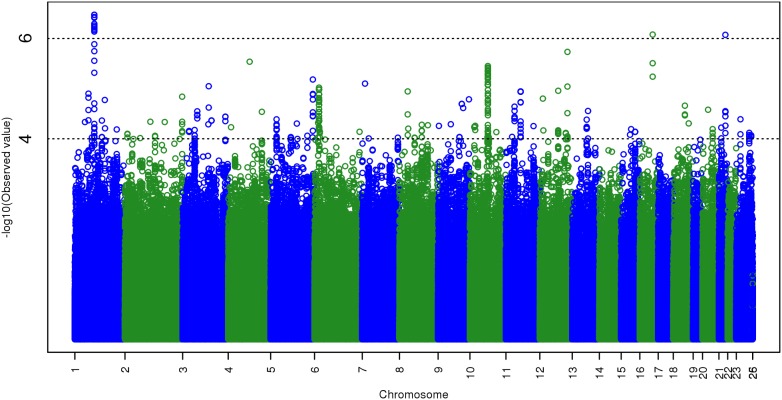
Manhattan plot of GWAS *p*-values, all 2,546,647 observed and imputed SNPs. Chromosome 23 = X chromosome, chromosome 25 = pseudoautosomal region of sex chromosome. Chromosome 26 indicates mitochondrial DNA. SNPs are plotted by serial position on each chromosome. Genome-wide significance is –log_10_(*p*) >7.30, which no SNP reaches. The peak on chromosome 1 is due to 11 SNPs (rs10922924, rs3856228, plus 9 others imputed nearby) that span about 14 kb not within a known gene. The peaks on chromosomes 16 and 21 are each due to a single imputed SNP, respectively rs16947526 and rs9982370.

**Figure 2:**
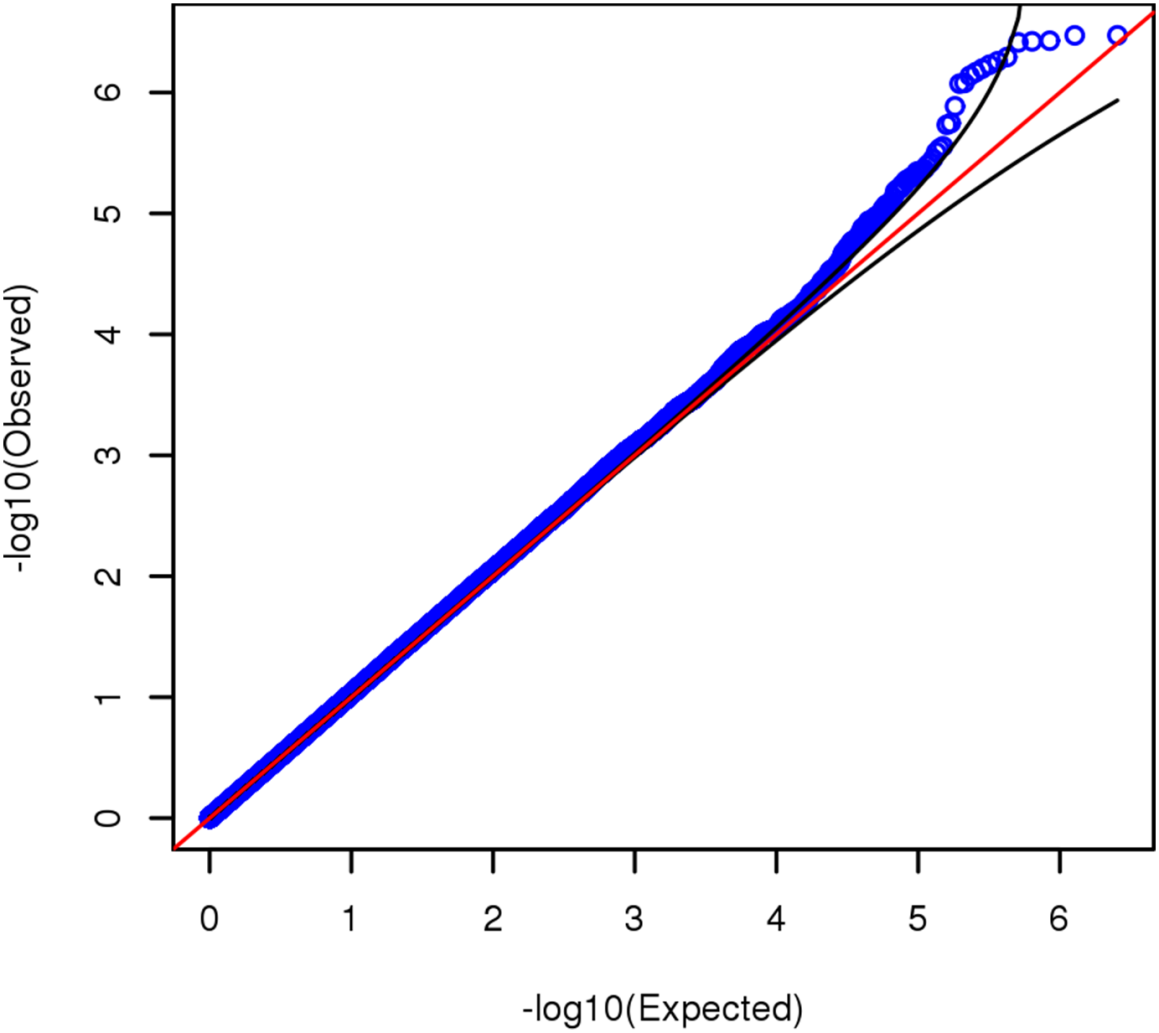
Uniform quantile-quantile plot of GWAS *p*-values, all 2,546,647 observed and imputed SNPs. The black curves delineate 95% confidence limits.

Estimates of the fixed and random effects from the covariates-only FGLS regression are presented in Table S2. *P*-values from the GWAS are depicted in Figures 1, 2, S1, and S2. Figure 1 is a “Manhattan plot” of the GWAS *p*-values from the 2,546,647 observed and imputed SNPs. The *y*-axis of a Manhattan plot is –log_10_(*p*). The *x*-axis is divided into chromosomes, and within each chromosome, SNPs are ordered by base-pair position. Chromosomes above #22 refer to different parts of the sex chromosomes and to mitochondrial DNA (see figure captions). No SNPs reached genome-wide significance, which in this metric would be –log_10_(*p*) >7.30. The association signal exceeding 6 on chromosome 1 is due to 11 SNPs (9 imputed) spanning about 14 kb, not within a known gene. The signal exceeding 6 on chromosome 16 is due to a single imputed SNP in the *FA2H* gene, rs16947526, of borderline imputation quality (*R* ^2^ = 0.52). On chromosome 21, the signal exceeding 6 is due to a single imputed SNP in the *ERG* gene, rs9982370. When only the observed SNPs are plotted (Figure S1), the only elevation above 6 occurs on chromosome 1.

Under the null hypothesis, *p*-values are uniformly distributed on interval (0, 1). Figures 2 and S2 are uniform quantile-quantile (QQ) plots of the GWAS *p*-values from, respectively, the 2,546,647 observed and imputed SNPs, and the 527,829 observed SNPs only. Under the null hypothesis, *p*-values from independent statistical tests are expected to follow the diagonal red line. Both QQ plots show some divergence from the null distribution, where the observed *p*-values tend to be more extreme than expected. To quantify this deviation, we can convert the *p*-values to quantiles from a chi-square distribution on 1 *df*, and compare their median and mean to the null values of 0.455 and 1, respectively. The ratio of the observed to the expected median is known as the genomic inflation factor, λ [53]. When this is done with observed and imputed SNPs together, median = 0.475 (λ = 1.044) and mean = 1.037; when done with observed SNPs only, the median = 0.471 (λ = 1.035) and mean = 1.031. This departure from the null may indicate massively polygenic inheritance of FSIQ, wherein few if any SNPs yield genome-wide significant association signals, but the overall distribution of test statistics reflects the presence of a large number of nonzero effects [54].

There are clearly some *p*-values that lie outside the confidence limits in Figures 2 and S2. However, because of LD among SNPs, the assumption of independent statistical tests is violated to begin with, and so one extreme result usually carries others with it. It stands to reason that this effect of LD would be more pronounced when imputed SNPs are included, since imputation methods rely on the LD (correlation) structure among SNPs to achieve denser coverage of the genome.

Our statistical inference from FGLS regression assumes that families’ vectors of residuals follow a multivariate-normal distribution in the population. If this assumption is met, then family members’ residuals will be marginally distributed as univariate normal, and the squared Mahalanobis distance from the origin of families’ residual vectors will be distributed as chi-square. Figures S3 and S4 present QQ plots that respectively check the observed distributions of individual residuals and family Mahalanobis distances against their theoretical distributions. The plots do not show severe departures from the theoretical distributions (though logically, these checks can only disconfirm, and not confirm, multivariate normality). The departure from normality evident in Figure S3 likely reflects that the far lower tail of the population IQ distribution is poorly represented in our sample.

### *VEGAS*

**Figure 3:**
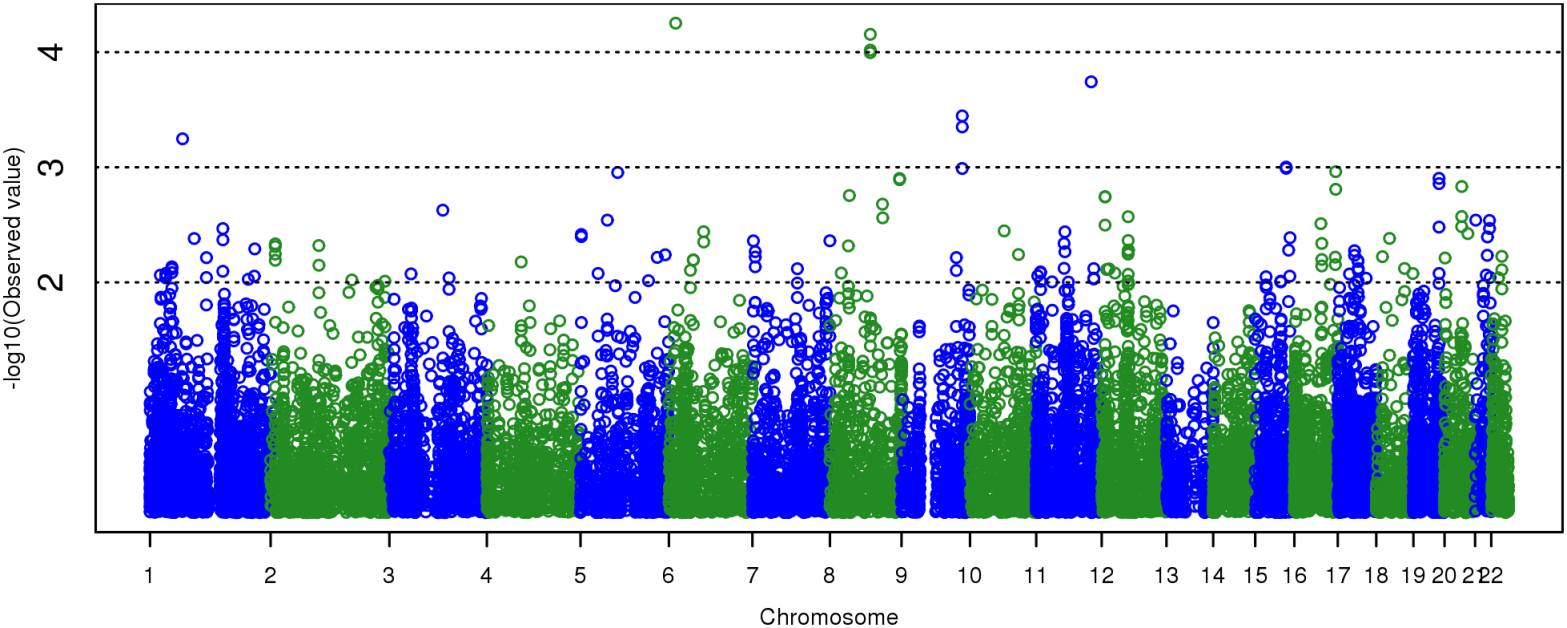
Manhattan plot for gene-based *p*-values from *VEGAS*. Analysis input was GWAS *p*-values from 2,485,149 autosomal SNPs, both observed and imputed. Abscissa position of each point is the gene’s beginning base-pair position, NCBI genome build 36. Genome-wide significance is –log_10_(*p*) >5.55, which no gene reaches.

**Figure 4:**
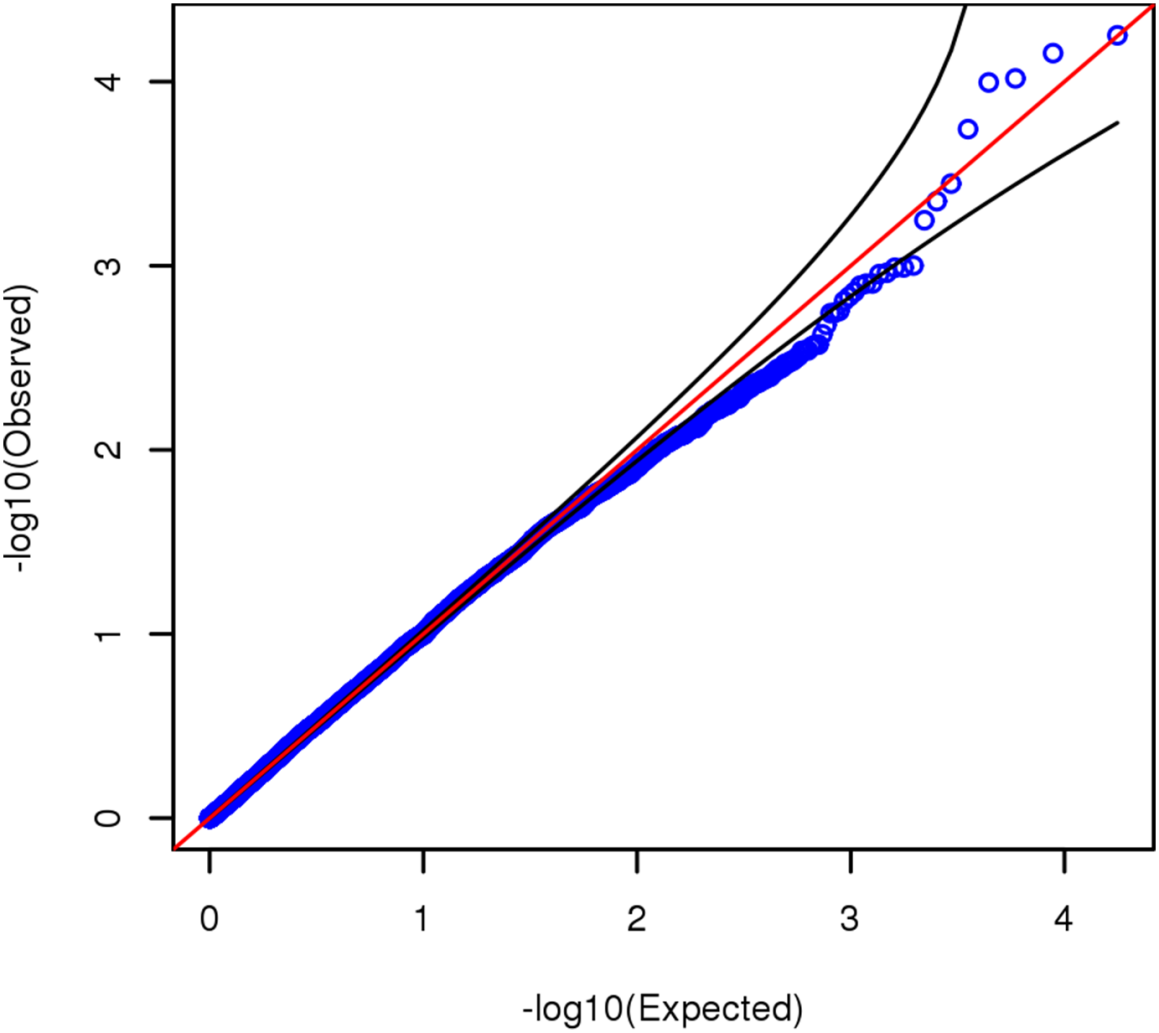
Uniform quantile-quantile plot for gene-based *p*-values from *VEGAS*. Analysis input was GWAS *p*-values from 2,485,149 autosomal SNPs, both observed and imputed. Black curves delineate 95% confidence limits.

The resulting gene-based *p*-values from *VEGAS* (inputting 2,485,149 autosomal SNPs, both observed and imputed) are depicted in Figure 3, a Manhattan plot, and Figure 4, a QQ plot. Figure 4 suggests that *VEGAS* has a somewhat conservative bias in these data. No gene in Figure 3 reaches the genome-wide significance level recommended for *VEGAS*, which in this metric would be –log_10_(*p*) >5.55. As shown in Figures S5 and S6 (results when inputting only 515,385 genotyped autosomal SNPs), our conclusions would be substantially unchanged if we had restricted our analyses to observed SNPs only. Our data do not support association of *FNBP1L* with GCA (*p* = 0.727).

### Polygenic scoring

**Figure 5:**
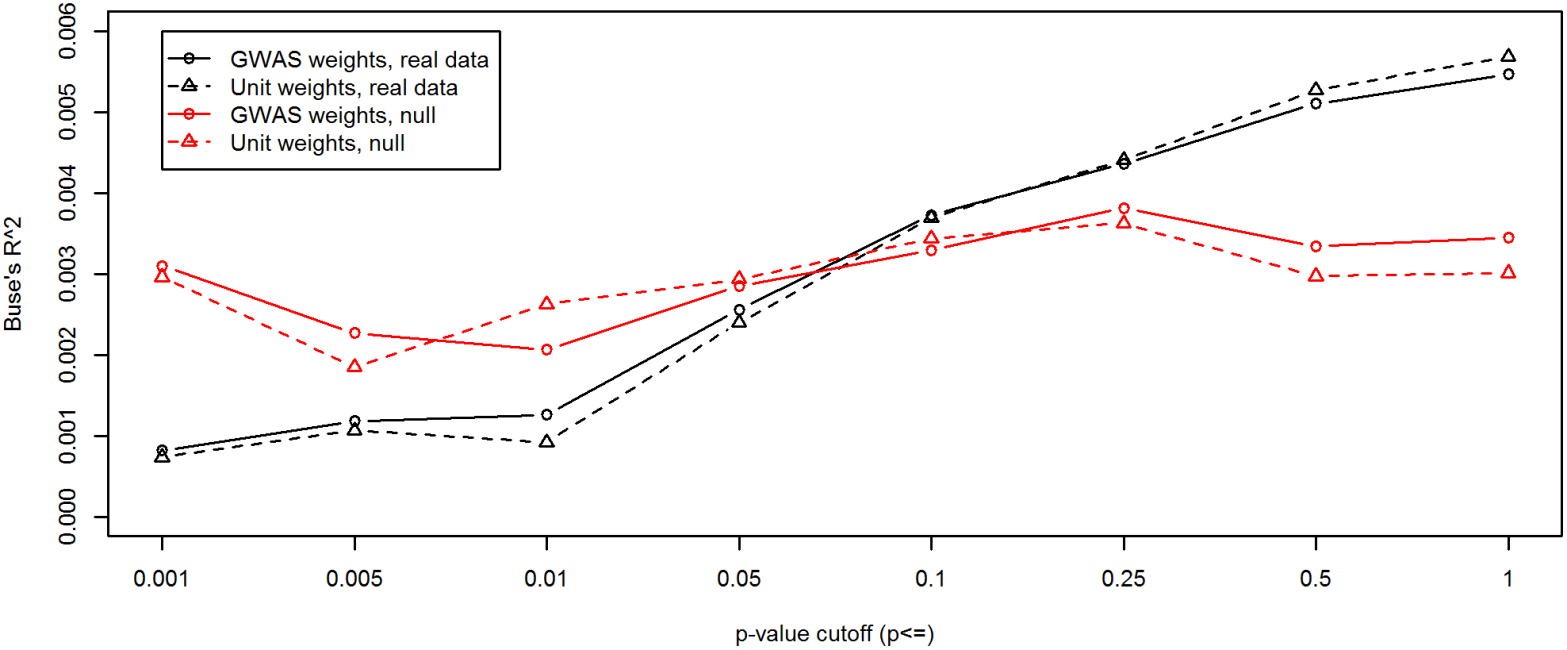
Five-fold cross-validation *R* ^2^ of polygenic score (averaged across subsamples, in black) for predicting FSIQ residualized for covariates, compared to results from simulated null data (98^th^ percentiles, in red). Black lines represent cross-validation Buse’s *R* ^2^ [51] for predicting residualized FSIQ, averaged across the 5 subsamples. In each subsample, residualized FSIQ was predicted from polygenic score calculated from regression weights obtained in the other 4 subsamples. “*P*-value cutoff” dictated how small a SNP’s *p*-value had to be in the calibration GWAS to be included in calculating polygenic score for the validation sample. Red lines represent the results of 50 iterations of parametric bootstrapping, which conducted polygenic scoring under cross-validation using data simulated under the null of independence between phenotype and SNP genotypes (conditional on covariates). Each point plotted for the red lines is the 98^th^ percentile, among the 50 iterations of parametric bootstrapping, of *R* ^2^ at that *p*-value cutoff. Polygenic score was either calculated directly from the GWAS weights (solid lines) or from signed unit weights (dashed lines; see text).

Figure 5 depicts cross-validation performance (Buse’s *R* ^2^) of polygenic score, averaged across subsample, and plotted in black by *p*-value cutoff and weighting scheme (in Figure S7, the *R* ^2^s from each subsample are plotted as separate trendlines). The red lines in Figure 5 depict the 98^th^ percentiles of Buse’s *R* ^2^, among the 50 iterations of parametric bootstrapping under the null, for each combination of *p*-value cutoff and weighting scheme. One notable result here is that the polygenic score, when calculated from signed unit-weighted SNPs, performed about as well as when it was calculated from the actual single-SNP GWAS regression weights. Another result evident in Figure 5 is the trend in cross-validation performance across *p*-value cutoffs: the predictive accuracy is maximized when using all genotyped SNPs (with best *R* ^2^ around 0.55%). This conclusion is further supported by comparing the black and red lines, which indicates that the “signal” in the real data was only reliably distinguishable from simulated “noise” at lenient *p*-value cutoffs.

### *GCTA*

At Yang et al.’s [29] suggested genetic-relatedness ceiling of 0.025 in our dataset, *N* = 3,322 of our participants were included in analysis, yielding 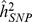 = 0.35 (*SE* = 0.11). Figure S8 presents normal QQ plots of individuals’ total genetic-effect eBLUPs and residuals. These plots resemble those of the FGLS residuals (Figure S3). However, the QQ plot of the eBLUPs may not be very informative about the true distribution of the random effects, since the eBLUPs were computed from a model that assumes normality in the first place, and the observed distribution of BLUPs can greatly depend upon the random effects’ assumed theoretical distribution (e.g., Ref [55]).

## Discussion

The present study is a “GWAS Plus” for general cognitive ability, conducted in a sample of over 7,000 Caucasian participants from two longitudinal family studies. We conducted the GWAS *per se* using 2,546,647 SNPs: 527,829 from the Illumina 660W–Quad array, plus 2,018,818 imputed with reasonable reliability (imputation *R* ^2^ >0.5). The “Plus” in “GWAS Plus” refers to our additional analyses that involve predicting the phenotype from more than one SNP at a time. These analyses were (1) gene-based association tests in *VEGAS*, (2) polygenic scoring with five-fold cross-validation, and (3) a genomic-relatedness restricted maximum-likelihood analysis in *GCTA*.

Our least interesting results were from *VEGAS* (Figures 3 and 4). What *VEGAS* essentially does is test whether all SNP *p*-values in a gene significantly differ in distribution from the null. No gene achieved genome-wide significance (*p*<2.8×10^−6^ or –log_10_(*p*) >5.55), and the method appears to be slightly conservatively biased in our dataset, possibly because of differences between our actual LD structure and that of *VEGAS*’ reference dataset, HapMap CEU. Running *VEGAS* with LD estimated from data is possible, but it seems doubtful that the LD misspecification could be so severe as to suppress a robustly significant association signal. Certainly the most *a priori* plausible gene, *FNBP1L*, is not supported in our sample (*p* = 0.727).

Polygenic scoring is another way of combining the predictive power of multiple SNPs. At best, the polygenic score could predict 0.7% of variance in our analyses (Figure S7), which occurred when calculating the score from all genotyped SNPs. Presumably, better results could be obtained at stricter *p*-value cutoffs when the calibration sample is larger. Interestingly, our cross-validation analysis showed that signed unit SNP weights performed about as well as GWAS regression weights. This suggests that, at least when the calibration sample is relatively small, there is negligible loss in predictive accuracy when fixing all SNP effects to the same absolute magnitude, and using GWAS merely to determine the direction of each SNP’s effect. We attempted the unit-weighting to strike a different balance between bias and variance. The GWAS regression weights, while unbiased, are estimated with considerable sampling error. On the other hand, unit weights are presumably biased, but possibly less variable over repeated sampling. In fact, unit weights can rival optimal least-squares weights in terms of predictive accuracy, especially when the overall amount of predictive error is large [56].

We are somewhat surprised at the relative performance of the polygenic score at inclusive vis-à-vis exclusive *p*-value cutoffs. We expected that the peak would occur at a relatively stringent cutoff, and that most SNPs with *p* greater than 0.25 or so would be irrelevant noise. Peak *R* ^2^ occurred at stricter cutoffs for the three replication cohorts of Benyamin et al. [33], including the one from MCTFR (at *p*≤0.01), which is a subsample of the present study sample. Likewise, in Lango Allen et al.’s [25] report on height, the average *R* ^2^ across five validation samples was highest at *p*≤0.001. However, both Benyamin et al. and Lango Allen et al. had the advantage of larger calibration samples than we did here. With larger calibration samples, estimates of SNP weights have less sampling error, and a given non-null effect size corresponds to a smaller expected *p*-value in the calibration sample. Evidently, our most significant SNPs had limited predictive power, but a heap of non-significant SNPs can better contribute to prediction in the aggregate. Polygenic scores calculated from all 527,829 genotyped SNPs at best account for about 0.7% of phenotypic variance, a value that contrasts sharply with parameter estimates from *GCTA*, even though both represent the proportion of variance attributable to every SNP on the array.

No single SNP has yet been replicably associated with human intelligence at genome-wide significance levels, and our GWAS results do not change that fact. This is not surprising, though, in light of our GWAS’ limited power. Given a conservative estimate of our effective sample size, we would have slightly above 80% power to detect a SNP accounting for 2% of phenotypic variance, which constitutes rather poor power. Even given an aggressive estimate of effective sample size, we would have slightly above 80% power to detect a SNP accounting for 0.6% of variance. But if realistic effect sizes are even smaller, like on the order of 0.2% to 0.4%[18], [19], this would still be inadequate. Needless to say, the major limitation of the present study was its limited sample size and commensurately limited power.

Even though we lacked sufficient power to detect a realistic SNP effect at genome-wide significance levels, the overall distribution of our test statistics and *p*-values differs slightly but appreciably from the null. This kind of genomic inflation can reflect population stratification(e.g., Ref [57]), but as shown analytically, through simulation, and through analysis of data from the GIANT Consortium [54], such genomic inflation is expected when there is no lurking population structure and the number of causal SNPs is large. Population stratification is doubtful in our case, because we carefully ensured that all our participants are White, and included 10 principal components from EIGENSTRAT as covariates. Even so, we cannot rule it out completely, so we cautiously interpret our observed genomic inflation as evidence of the massive polygenicity of GCA.

We regard our *GCTA* results as the most impressive and informative. The performance of our polygenic score at inclusive *p*-value cutoffs, plus the genomic inflation evident in our GWAS, suggest that there is a very large number of trait-relevant polymorphisms, each with a very small individual effect on FSIQ. Our results from *GCTA*–which were similar to those of earlier studies [32], [33]–provide even stronger evidence that this is so. We surmise that few behavior geneticists, once they understood the GREML method, were surprised that a substantial proportion of variance in cognitive ability and in height [58] could be attributed to genotyped SNPs on a chip. But, that is precisely why *GCTA* is so monumental: it has furnished molecular genetics with the result that quantitative genetics has predicted for decades, in support of the classical theory of polygenic inheritance. We see now how truly R. A. Fisher wrote when he penned these words in 1918 [59]: “the statistical properties of any feature determined by a *large number* of Mendelian factors have been successfully elucidated…In general, the hypothesis of cumulative Mendelian factors seems to fit the facts very accurately” (p. 432–433, emphasis supplied).

Readers may wonder at the discrepancy between the proportions of variance explainable by polygenic scoring from all genotyped SNPs and by *GCTA*, even though both methods attempt to use SNPs to account for phenotypic variance. The discrepancy is explainable by important differences between the two methods [31]. The essential reason is that the performance of polygenic scoring depends upon accurate calibration of many SNP weights, whereas the performance of GREML methods does not. The multiple SNP weights used to compute the polygenic score are estimated with sampling error, which error is expected to decrease its validation *R* ^2^. In contrast, GREML does not predict the phenotype from a linear composite of weighted genotypes. Instead, it estimates the extent to which genetic similarity among participants corresponds to their phenotypic similarity, based on the same principle as biometric analysis in, say, a twin study. It differs from biometric modeling in that it uses genome-wide marker data to calculate genetic similarity between participants who are not closely related, instead of relying on the expected genetic similarity between biological relatives according to quantitative-genetic theory. Visscher et al. [31] discuss the contrast between polygenic scoring and the GREML method, commenting that “the accuracy of prediction from estimated SNP effects can be very different from the proportion of variance explained in the population by those effects” (p. 524).

*GCTA* provided us with an 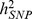 estimate of 35%, within the range of GREML effect sizes previously observed for cognitive ability [32], [33]. But, biometrical heritability estimates for GCA are typically in the range of 50% to 70%. This outcome, that through GREML methods common SNPs on a genome-wide array can account for most but not all of the heritability of a trait, also appears typical for cognitive ability, and for that archetypal polygenic quantitative trait, height [58]. This is known as the problem of “hidden heritability”[60]. Of course, biometrical analysis and GREML each estimate different quantities: 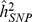 is only a lower-bound estimate of a phenotype’s additive heritability. What, then, might be the molecular basis for the heritability that is not captured by GREML estimates? Since 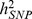 represents the proportion of phenotypic variance attributable to common SNPs on the array (and variants in tight LD with them), it stands to reason that the hidden heritability might be due to polymorphisms that are not common, or are not SNPs (such as copy-number variants), or are not tagged in the population by common SNPs. In any event, if specific polymorphisms underlying variation in GCA are to be discovered, gargantuan sample sizes, such as in the GIANT Consortium [25], will be necessary. But in the meantime, we can conclude that there are a great many unspecified polymorphisms associated with GCA, each with a very small effect–general cognitive ability is indeed “heritable [and] highly polygenic” (Ref [35], p. 1). The trait-relevant SNPs are each Lilliputian in effect size, but together, are legion in number.

## Supporting Information

Figure S1 Manhattan plot of GWAS *p*-values from 527,829 observed SNPs only. Chromosome 23 = X chromosome, chromosome 25 = pseudoautosomal region of sex chromosome. Chromosome 26 indicates mitochondrial DNA. SNPs are plotted by serial position on each chromosome. Genome-wide significance is -log_10_(*p*) >7.30, which no SNP reaches.
(TIF)

Figure S2 Uniform quantile-quantile plot for GWAS *p*-values from 527,829 observed SNPs only. The black curves delineate 95% confidence limits.
(TIF)

Figure S3 Normal quantile-quantile plots of FGLS residuals, graphed separately by family member. Plotted residuals were obtained from the covariates-only regression. The number of points in each plot is provided in the *y*-axis label. If families’ residual vectors are multivariate-normal in the population, then family members’ residuals are expected to be marginally univariate-normal. It can be seen that univariate normality provides a reasonably good approximation, except for some divergence in the lower tail.
(TIF)

Figure S4 Chi-square quantile-quantile plots of squared Mahalanobis distances (from the origin) of families’ FGLS residual vectors, graphed separately by family size.
(PDF)

Figure S5 Manhattan plot for gene-based *p*-values from *VEGAS*, inputting observed SNPs only. Analysis input was GWAS *p*-values from 515,385 autosomal SNPs on the Illumina array. Abscissa position of each point is the gene’s beginning base-pair position, NCBI genome build 36. Genome-wide significance is –log_10_(*p*) >5.55, which no gene reaches.
(TIF)

Figure S6 Uniform quantile-quantile plot for gene-based *p*-values from *VEGAS*, inputting observed SNPs only. Analysis input was GWAS *p*-values from 515,385 autosomal SNPs on the Illumina array. Black curves delineate 95% confidence limits.
(TIF)

Figure S7 Five-fold cross-validation of polygenic score, predicting FSIQ residuallized for covariates. Figure depicts cross-validation Buse’s *R* ^2^ for predicting residuallized FSIQ in the indicated subsample from polygenic score calculated from regression weights obtained in the other 4 subsamples. “*P*-value cutoff” dictated how small a SNP’s *p*-value had to be in the calibration GWAS to be included in calculating polygenic score for the validation sample. Polygenic score was either calculated directly from the GWAS weights (solid lines) or from signed unit weights (dashed lines; see text).
(TIF)

Figure S8 Normal quantile-quantile plots of predicted *GCTA* random effects. The left-hand panel depicts empirical best linear unbiased predictions (eBLUPs) of 3,322 participants’ total genetic effects, i.e. **g** in main-text Equation (4), conditional on the fixed effects. The right-hand panel depicts those participants’ residuals, given the fixed effects and the eBLUPs of **g**. As explained in the text, quantile-quantile plots of eBLUPs should be interpreted cautiously.
(TIF)

Table S1 Family patterns of GWAS data availability.
(DOCX)

Table S2 *RFGLS* parameter estimates for regression of FSIQ onto covariates only.
(DOCX)

Material S1Supplementary Appendix: 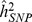 as function of genetic-relatedness cutoff. Includes Figures A1, A2, and A3(PDF)
