# Correction: Results of a “GWAS Plus:” General Cognitive Ability Is Substantially Heritable and Massively Polygenic

**DOI:** 10.1371/journal.pone.0121909

**Published:** 2015-03-23

**Authors:** The PLOS ONE Staff

There are several errors in mathematical expressions in the Introduction under the subheading GWAS Plus: GCTA. In the final sentence of the second paragraph, the expression for the distribution of u is incorrect. The subscript n should be an m and the subscript epsilon should be a u. The correct distribution is: $u∼N_{m}(0,I\sigma_{u}^{2})$. In the second sentence of the sixth paragraph, the expression for the distribution of g is incorrect. The subscript epsilon should be a g. The correct distribution is: $N_{n}(0,A\sigma_{g}^{2})$.
