# Supplementary figures and images for: Results of a “GWAS Plus:” General Cognitive Ability Is Substantially Heritable and Massively Polygenic
Source: PLoS One. 2014 Nov 10;9(11):e112390. doi: 10.1371/journal.pone.0112390 (PMC4226546; doi:10.1371/journal.pone.0112390)

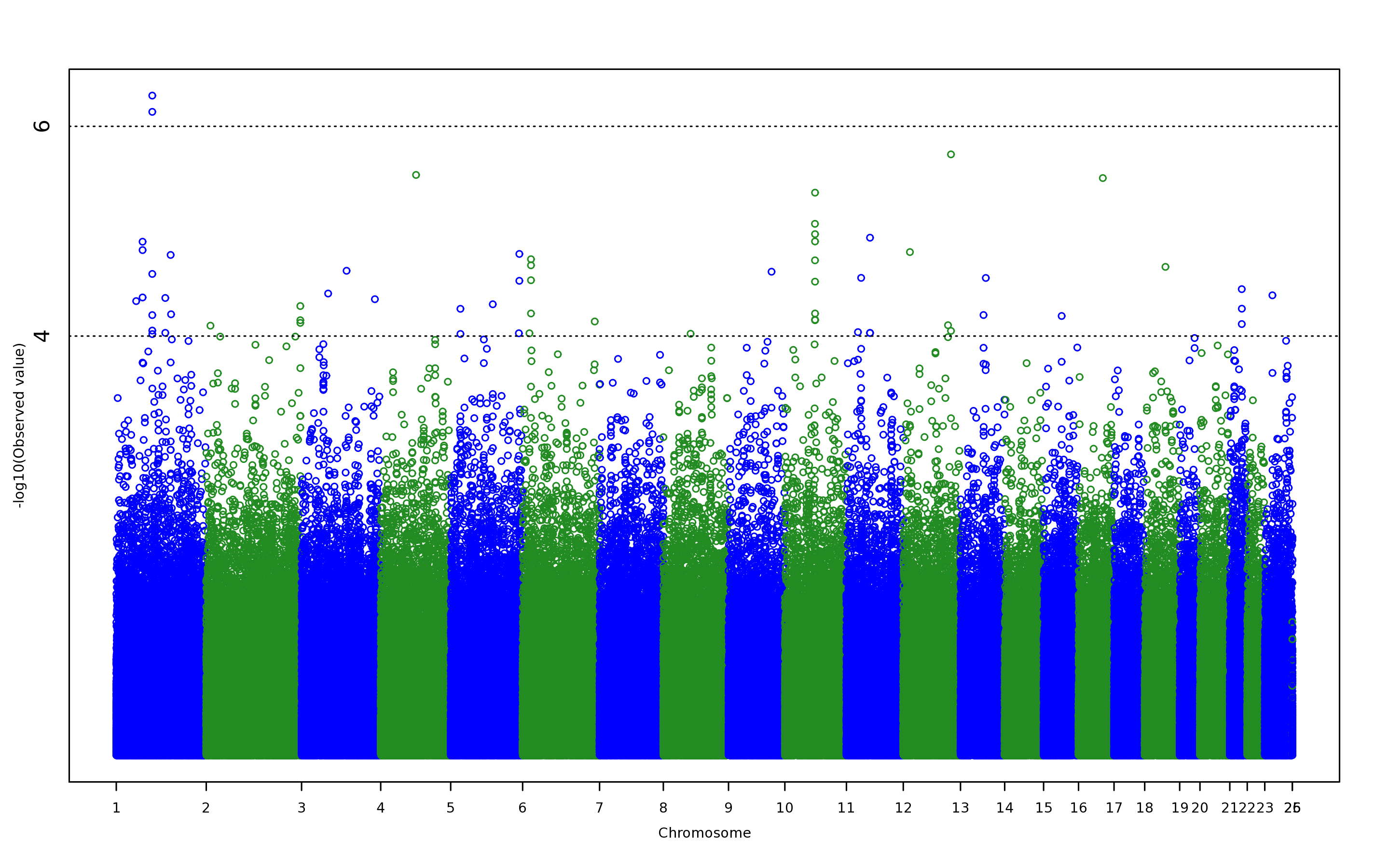

Supplement: Figure S1 — Manhattan plot of GWAS p-values from 527,829 observed SNPs only. Chromosome 23 = X chromosome, chromosome 25 = pseudoautosomal region of sex chromosome. Chromosome 26 indicates mitochondrial DNA. SNPs are plotted by serial position on each chromosome. Genome-wide significance is -log10(p) >7.30, which no SNP reaches. (TIF) [file pone.0112390.s001.tif]

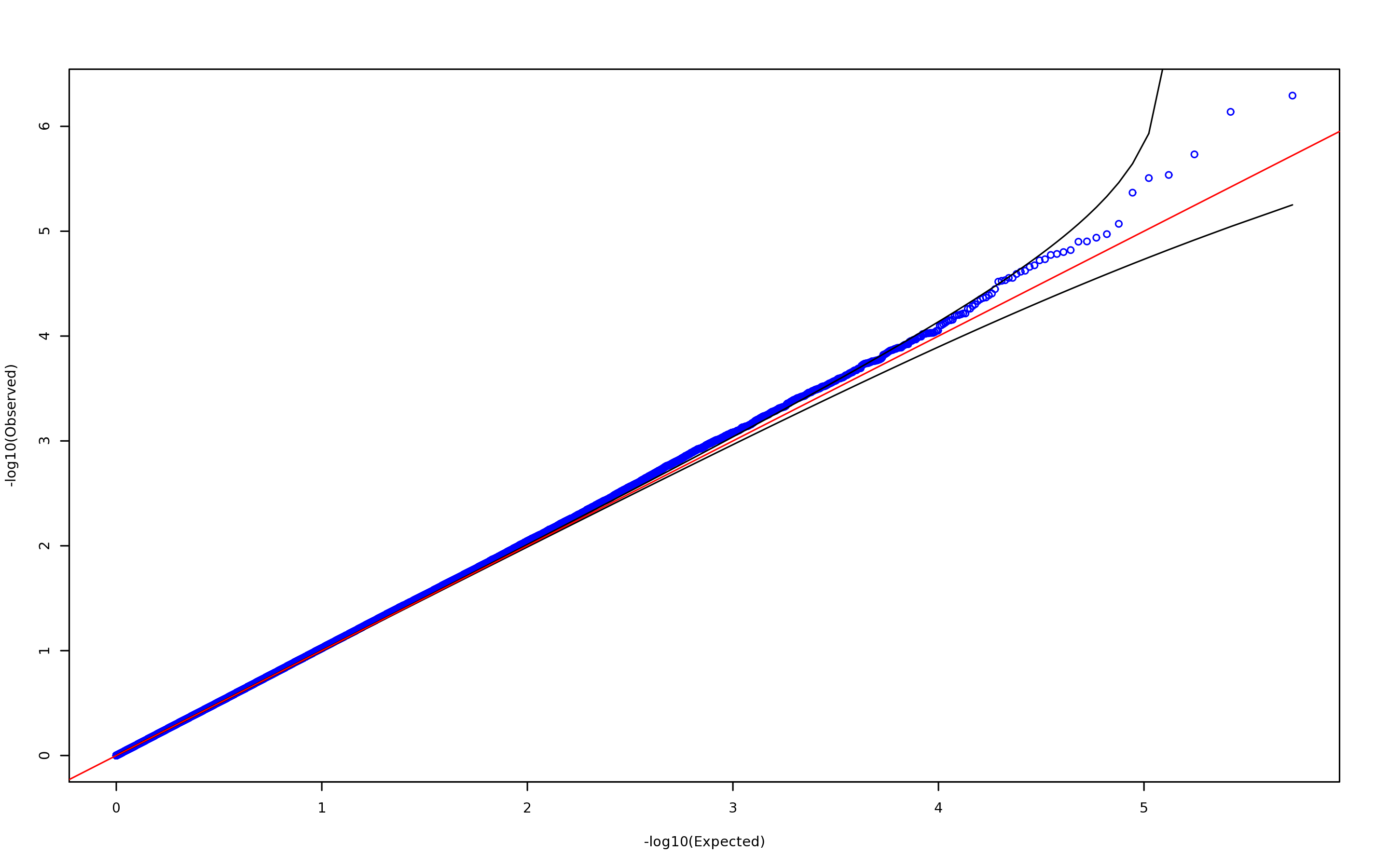

Supplement: Figure S2 — Uniform quantile-quantile plot for GWAS p-values from 527,829 observed SNPs only. The black curves delineate 95% confidence limits. (TIF) [file pone.0112390.s002.tif]

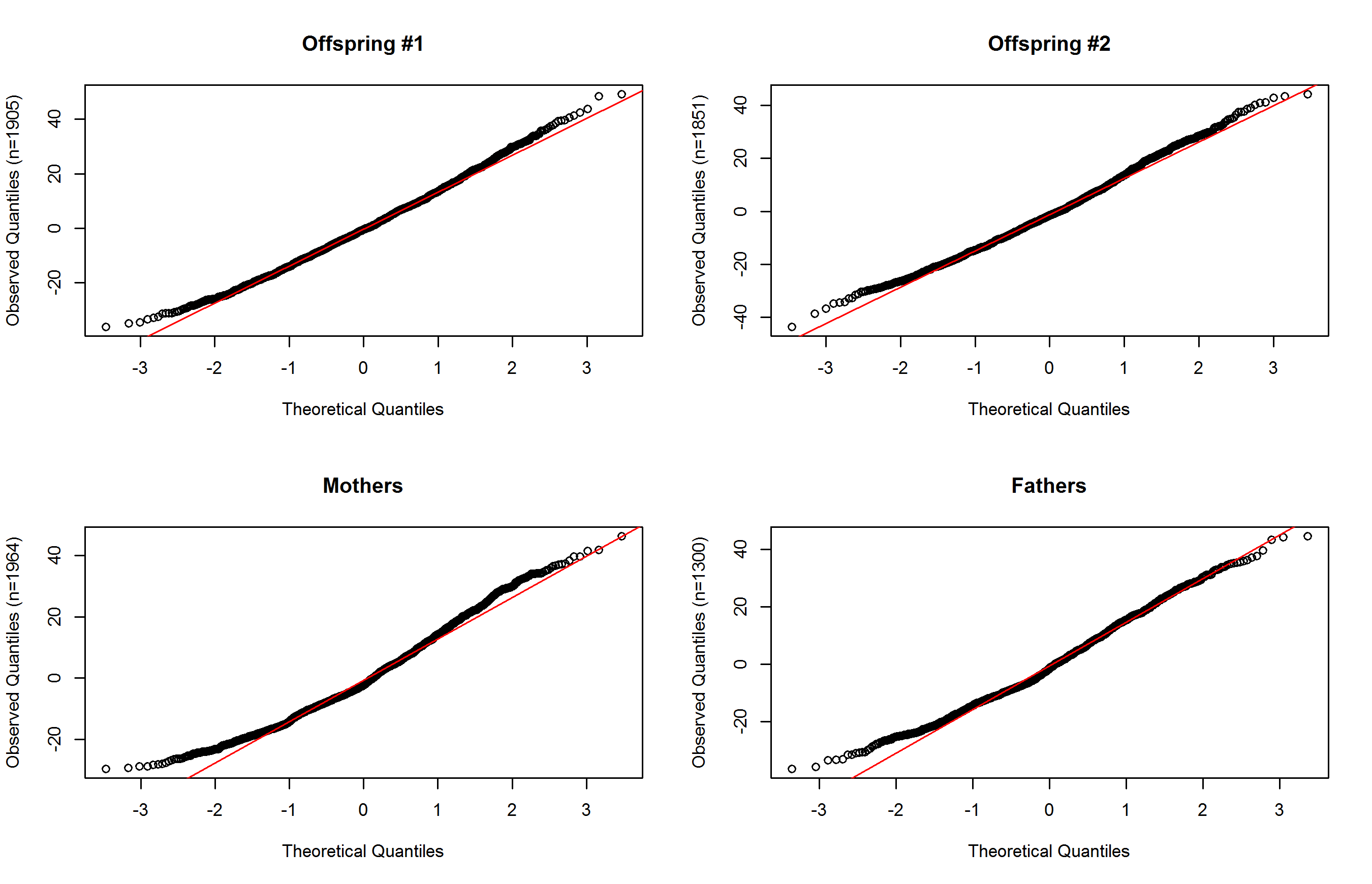

Supplement: Figure S3 — Normal quantile-quantile plots of FGLS residuals, graphed separately by family member. Plotted residuals were obtained from the covariates-only regression. The number of points in each plot is provided in the y-axis label. If families’ residual vectors are multivariate-normal in the population, then family members’ residuals are expected to be marginally univariate-normal. It can be seen that univariate normality provides a reasonably good approximation, except for some divergence in the lower tail. (TIF) [file pone.0112390.s003.tif]

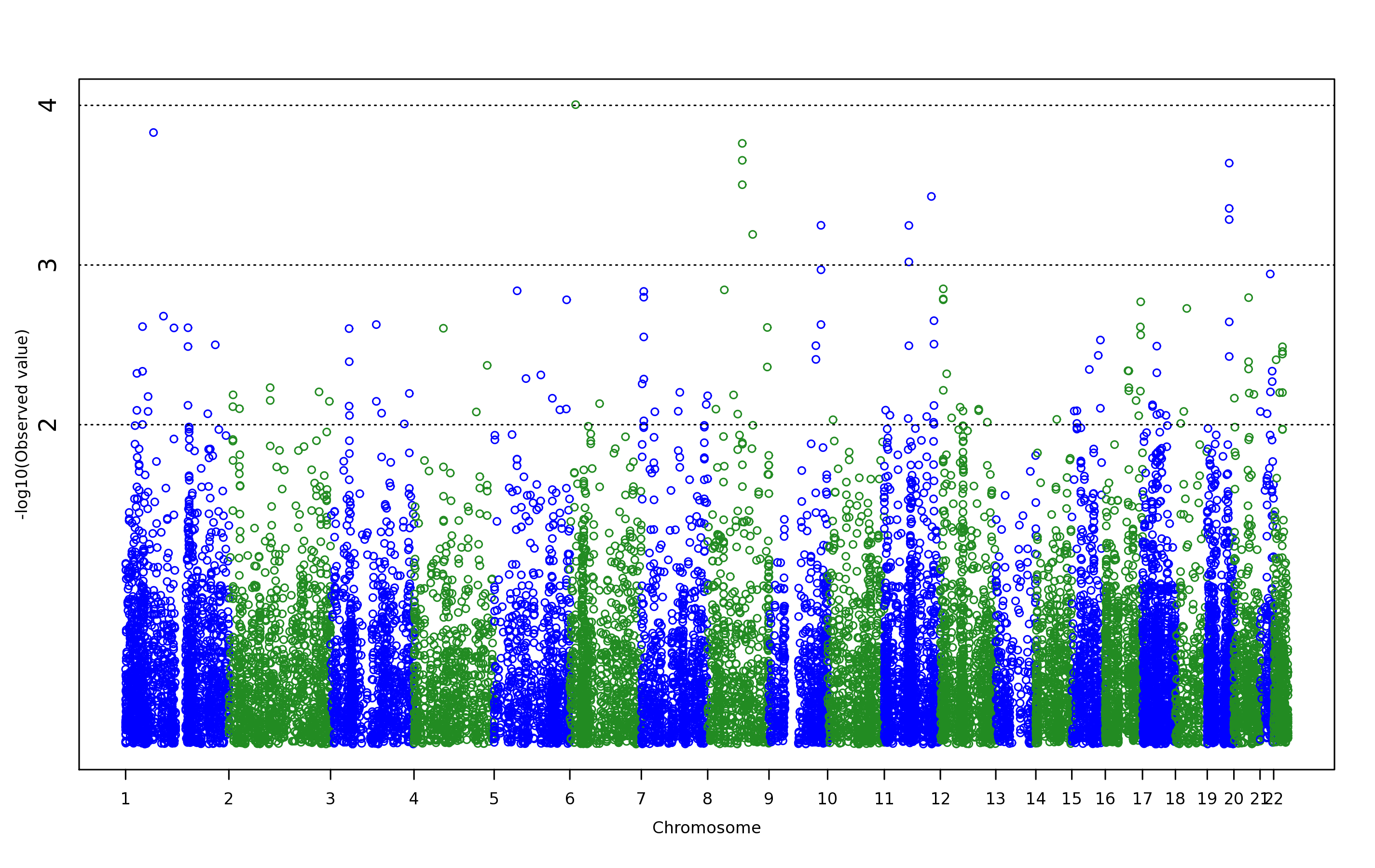

Supplement: Figure S5 — Manhattan plot for gene-based p-values from VEGAS, inputting observed SNPs only. Analysis input was GWAS p-values from 515,385 autosomal SNPs on the Illumina array. Abscissa position of each point is the gene’s beginning base-pair position, NCBI genome build 36. Genome-wide significance is –log10(p) >5.55, which no gene reaches. (TIF) [file pone.0112390.s005.tif]

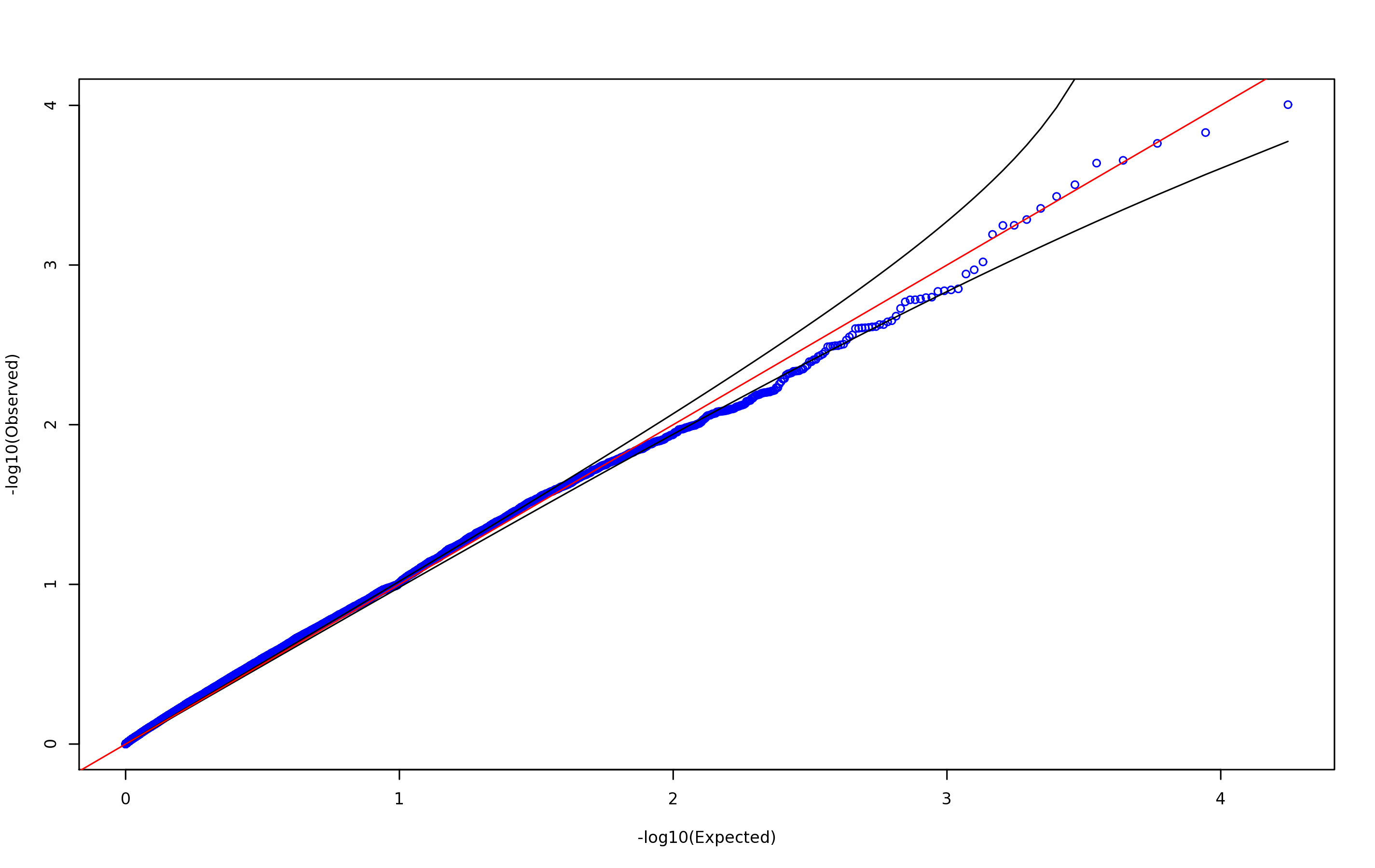

Supplement: Figure S6 — Uniform quantile-quantile plot for gene-based p-values from VEGAS, inputting observed SNPs only. Analysis input was GWAS p-values from 515,385 autosomal SNPs on the Illumina array. Black curves delineate 95% confidence limits. (TIF) [file pone.0112390.s006.tif]

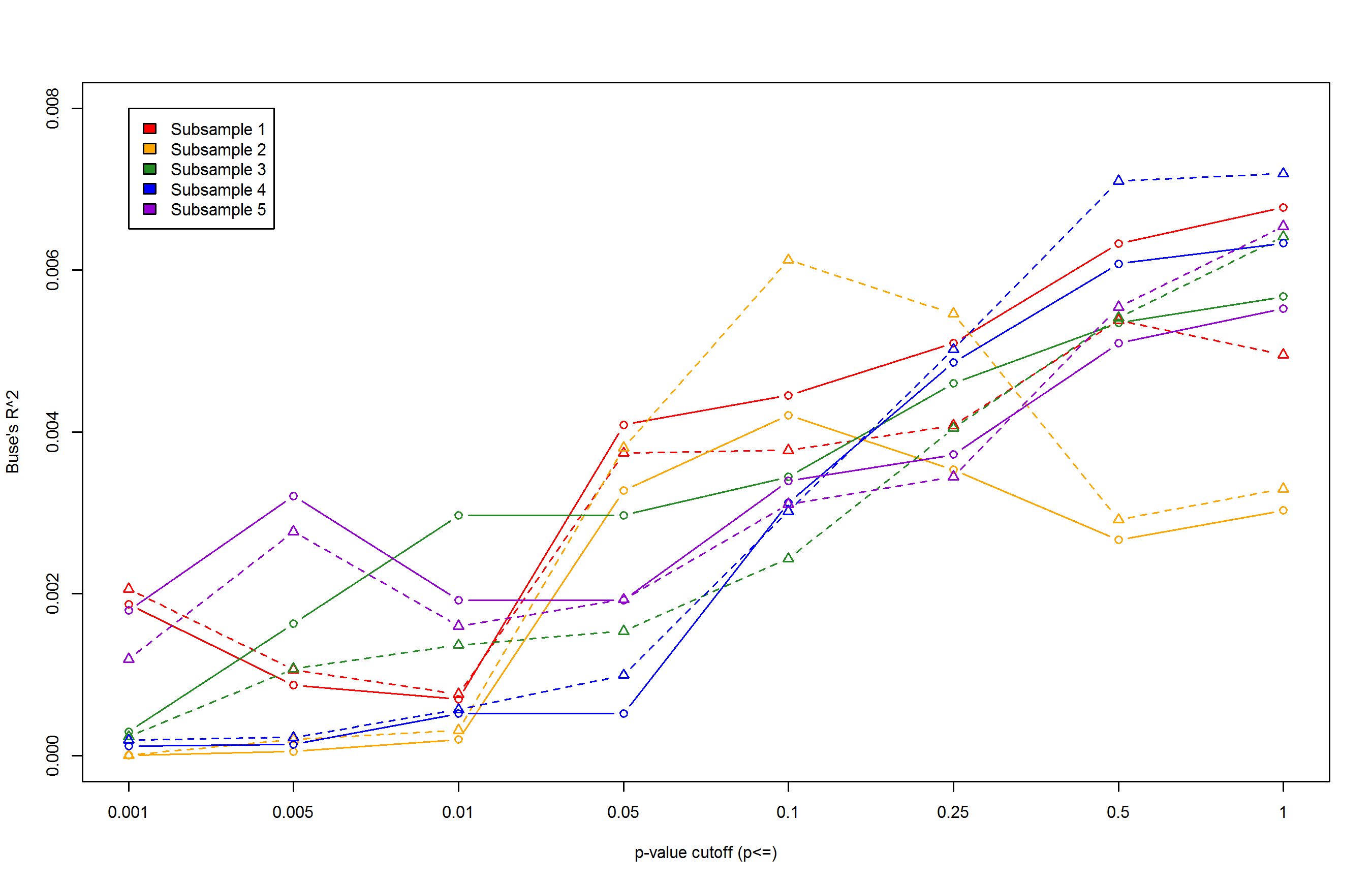

Supplement: Figure S7 — Five-fold cross-validation of polygenic score, predicting FSIQ residuallized for covariates. Figure depicts cross-validation Buse’s R 2 for predicting residuallized FSIQ in the indicated subsample from polygenic score calculated from regression weights obtained in the other 4 subsamples. “P-value cutoff” dictated how small a SNP’s p-value had to be in the calibration GWAS to be included in calculating polygenic score for the validation sample. Polygenic score was either calculated directly from the GWAS weights (solid lines) or from signed unit weights (dashed lines; see text). (TIF) [file pone.0112390.s007.tif]

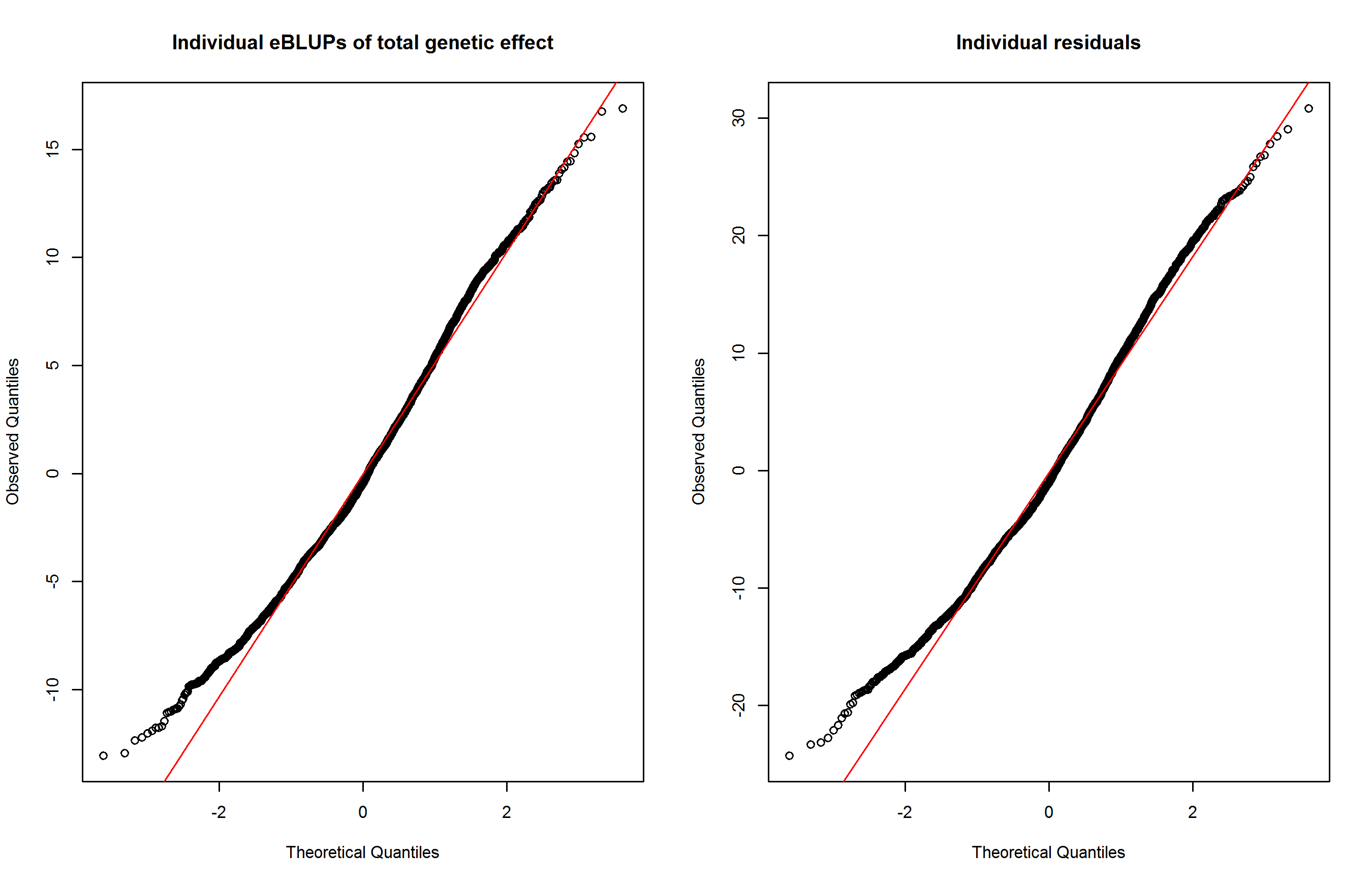

Supplement: Figure S8 — Normal quantile-quantile plots of predicted GCTA random effects. The left-hand panel depicts empirical best linear unbiased predictions (eBLUPs) of 3,322 participants’ total genetic effects, i.e. g in main-text Equation (4), conditional on the fixed effects. The right-hand panel depicts those participants’ residuals, given the fixed effects and the eBLUPs of g. As explained in the text, quantile-quantile plots of eBLUPs should be interpreted cautiously. (TIF) [file pone.0112390.s008.tif]
