# Supplementary material for: Results of a “GWAS Plus:” General Cognitive Ability Is Substantially Heritable and Massively Polygenic
Source: PLoS One. 2014 Nov 10;9(11):e112390. doi: 10.1371/journal.pone.0112390 (PMC4226546; doi:10.1371/journal.pone.0112390)
Supplement: Figure S4 — Chi-square quantile-quantile plots of squared Mahalanobis distances (from the origin) of families’ FGLS residual vectors, graphed separately by family size. (PDF) [file pone.0112390.s004.pdf]

Figure S4. Chi-square quantile-quantile plots of squared Mahalanobis distances (from the origin) of families' FGLS residual vectors, graphed separately by family size.

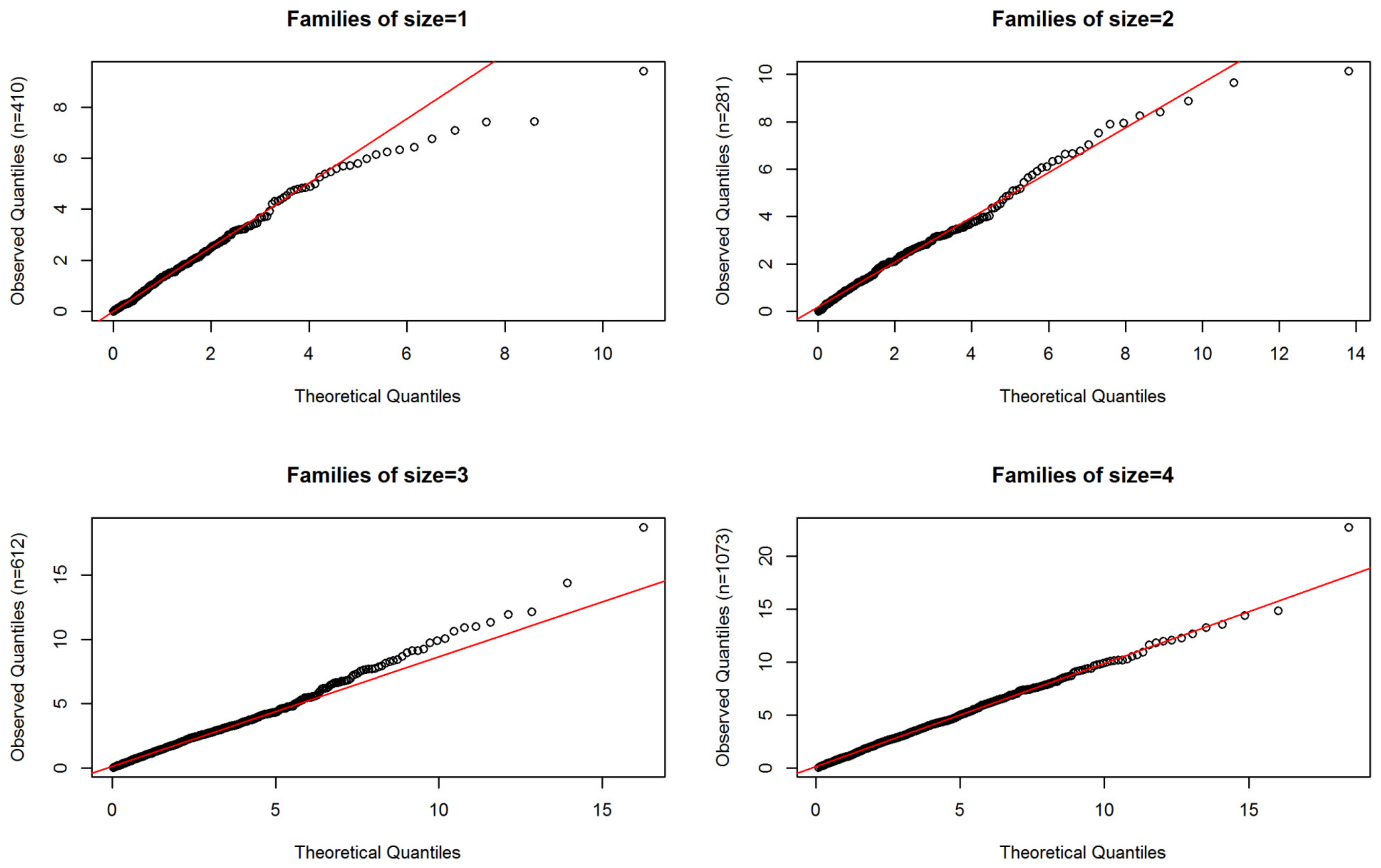

Let $\boldsymbol{\varepsilon}_i$ denote the $s_i \times 1$ column vector of FGLS residuals (from the covariates-only regression) of family i. Let $\hat{\boldsymbol{\Sigma}}_i$ denote the $s_i \times s_i$ symmetric block of the estimated residual-covariance matrix $\hat{\boldsymbol{\Sigma}}$ that corresponds to family i—basically, $\hat{\boldsymbol{\Sigma}}_i$ is the matrix giving the estimated residual-covariance structure for a family of the same type and composition of family i. Then, family i's squared Mahalanobis distance from the origin is $d_i^2 = \boldsymbol{\varepsilon}_i^T \hat{\boldsymbol{\Sigma}}_i^{-1} \boldsymbol{\varepsilon}_i$. If families' residual vectors are multivariate-normal, then variable d^2 is expected to be distributed as chi-square, with *df* equal to family size s_i. The number of points in each panel is provided in its *y*-axis label. It can be seen that the chi-square distribution is a reasonably good approximation to the data, except for a few outliers.
