# Supplementary material for: Results of a “GWAS Plus:” General Cognitive Ability Is Substantially Heritable and Massively Polygenic
Source: PLoS One. 2014 Nov 10;9(11):e112390. doi: 10.1371/journal.pone.0112390 (PMC4226546; doi:10.1371/journal.pone.0112390)
Supplement: Table S1 — Family patterns of GWAS data availability. (DOCX) [file pone.0112390.s009.docx]

Table S1. Family patterns of GWAS data availability.

| Availability Pattern | Frequency |
| --- | --- |
| OOMF | 1073 |
| OMF | 40 |
| OOM | 482 |
| OOF | 90 |
| OO | 130 |
| OM | 95 |
| OF | 16 |
| MF | 40 |
| O | 55 |
| M | 234 |
| F | 41 |
| Step-parents | 80 |

Table notes: “Frequency” is the number of families in the GWAS to which the corresponding availability pattern applied. Each availability pattern indicates which members of a four-person nuclear family had non-missing genotype and phenotype data: O = offspring, M = mother, F = father. For example, a family in which all four members had available data would be OOMF, whereas OM is for families from which only the mother and one child could be included in analysis. Step-parents are treated as independent observations, as though each belonged to his/her own separate family unit.
