# Supplementary material for: Results of a “GWAS Plus:” General Cognitive Ability Is Substantially Heritable and Massively Polygenic
Source: PLoS One. 2014 Nov 10;9(11):e112390. doi: 10.1371/journal.pone.0112390 (PMC4226546; doi:10.1371/journal.pone.0112390)
Supplement: Table S2 — RFGLS parameter estimates for regression of FSIQ onto covariates only. (DOCX) [file pone.0112390.s010.docx]

Table S2. *RFGLS* parameter estimates for regression of FSIQ onto covariates only.

| Fixed Effects | | |  | Random Effects | | |
| --- | --- | --- | --- | --- | --- | --- |
| Parameter | Estimate | SE |  | Parameter | Estimate | SE |
| Intercept | 115.26 | 0.84 |  | Correlation, Spousal | 0.35 | 0.03 |
| Sex | -2.84 | 0.31 |  | Correlation, Bio Mother-Child | 0.44 | 0.02 |
| Birth Year | -0.09 | 0.01 |  | Correlation, Bio Father-Child | 0.44 | 0.02 |
| PC1 | 0.11 | 0.14 |  | Correlation, MZ twin | 0.80 | 0.01 |
| PC2 | -0.24 | 0.13 |  | Correlation, Full Siblings | 0.49 | 0.03 |
| PC3 | -0.11 | 0.13 |  | Correlation, Adopt Mother-Child | 0.11 | 0.12 |
| PC4 | -0.04 | 0.13 |  | Correlation, Adopt Father-Child | 0.44 | 0.24 |
| PC5 | -0.13 | 0.13 |  | Correlation,  Adoptive Siblings | 0.31 | 0.11 |
| PC6 | -0.24 | 0.13 |  | Variance,  Offspring | 194.34 | 5.34 |
| PC7 | 0.17 | 0.13 |  | Variance,  Mothers | 185.56 | 5.92 |
| PC8 | 0.11 | 0.13 |  | Variance, Fathers | 212.95 | 8.33 |
| PC9 | 0.23 | 0.13 |  | Variance,  Stepparents | 265.99 | 43.43 |
| PC10 | -0.12 | 0.13 |  |  |  |  |

Table notes: Covariates were birth year (2 digits), dummy variable for female sex, and the first 10 EIGENSTRAT principal components. The fixed effects are regression coefficients. The random effects are the parameters *RFGLS* uses to assemble the residual covariance matrix. *RFGLS* provides two specifications for the residual covariance matrix: (1) a variance-component specification, using an additive-genetic, shared-environmental, and unshared-environmental component; and (2) an “unstructured” specification, using the 12 random effects presented above. We compared Akaike’s Information Criterion (*AIC*) from the covariates-only regression under the variance-component specification (*AIC* = 42695.29) and the unstructured specification (*AIC* = 42565.96), with the latter being preferred.
