# Supplementary material for: Results of a “GWAS Plus:” General Cognitive Ability Is Substantially Heritable and Massively Polygenic
Source: PLoS One. 2014 Nov 10;9(11):e112390. doi: 10.1371/journal.pone.0112390 (PMC4226546; doi:10.1371/journal.pone.0112390)
Supplement: Material S1 — Supplementary Appendix: as function of genetic-relatedness cutoff. Includes Figures A1, A2, and A3 (PDF) [file pone.0112390.s011.pdf]

Supplementary Appendix: $\hat{h}^2_{SNP}$ as function of genetic-relatedness cutoff

The GREML software *GCTA* allows the user to set a maximum allowable degree of genetic relatedness among participants entered into analysis (herein, the "genetic-relatedness ceiling"). To gain further insight into the quantity estimated by *GCTA*, h^2_{SNP}, we conducted an exploratory analysis in which we calculated $\hat{h}^2_{SNP}$ at varying genetic-relatedness ceilings. Specifically, we ran *GCTA* with FSIQ as the phenotype and with the same covariates used in the GWAS (sex, birth year, 10 EIGENSTRAT principal components) as fixed effects, 234 times over. Each of the 234 runs used a different genetic-relatedness ceiling, ranging from 0.005 to 1.17, in increments of 0.005. As the ceiling increased, both the number of participants, and the degree to which participants in the analysis could be genetically related to one another, increased.

Figure A1 (below) graphs $\hat{h}^2_{SNP}$ as a function of genetic-relatedness ceiling, with error bars representing ±1 standard error. As the ceiling increased, more participants were included in the analysis, and the statistical precision of $\hat{h}^2_{SNP}$ increased. More importantly, the point estimate itself increased as well. Below a ceiling of 0.015, sample size was less than 300, and the software produced nonsensical negative point estimates. A noticeable spike in $\hat{h}^2_{SNP}$ is evident around 0.5, where full siblings (including DZ twins) were introduced. Something similar happens around 1.0, where the MZ twins were introduced. Figure A2 (below) shows how the sample size of the *GCTA* analysis increases with relatedness ceiling, and Figure A3 (below) shows $\hat{h}^2_{SNP}$ as a function of sample size rather than the ceiling. When we systematically incremented the ceiling by regular intervals, three sample-size plateaus were evident (Figure A2). For ceilings between 0.15 and 0.4, *N* and $\hat{h}^2_{SNP}$ were steady around 3,600 and 0.43, respectively. Between ceilings of 0.56 to 0.98, they were steady at about 6,050 and 0.66. When all 7,100 GWAS participants were included at a ceiling of 1.17, *GCTA* yielded $\hat{h}^2_{SNP}$ = 0.77 (SE = 0.01).

According to Yang et al. (Ref 28), if the purpose of *GCTA* is to estimate how much phenotypic variance is attributable to the common SNPs on a genome-wide array, then close relatives should be excluded from analysis. They suggest a genetic-relatedness ceiling of 0.025. The reason for excluding close relatives is that, if they are instead included, then the *GCTA* estimator may overestimate the actual variance attributable to the SNPs on the array. When close relatives are included, the *GCTA* estimator functions more like a pedigree-based estimator, which captures the influence of all trait-relevant polymorphisms that contribute to familial resemblance, no matter how rare, and not just the genotyped SNPs (and other polymorphisms the SNPs tag). In the extreme case, then, a *GCTA* variance component estimated from MZ twins could even reflect the influence of *de novo* mutations, which contribute to MZ-twin resemblance but are not tagged in the population by common SNPs. It would seem, then, that if one wants to estimate the *overall* heritability of a phenotype with *GCTA*, inclusion of close relatives is the way to go. It might be tempting to conclude that the $\hat{h}^2_{SNP}$ values in Figure A1 produced when the relatedness ceiling is above 1.0 are molecular-genetic estimates of the true, broad-sense heritability of GCA. But that is not necessarily the case: as Yang et al. (Ref 28) also remind us, including close relatives confounds genetic resemblance with shared-environmental influence.

Although $\hat{h}^2_{SNP}$ apparently increased with sample size (Figure A3), this was because both sample size and $\hat{h}^2_{SNP}$ increased as the genetic-relatedness ceiling—the parameter of the *GCTA* analyses we directly manipulated—was relaxed. We would not expect $\hat{h}^2_{SNP}$, which is unbiased,

to systematically increase with sample size when additional *unrelated* individuals are added to the sample (Visscher et al., Ref 30). In contrast, we would anticipate greater $\hat{h}^2_{SNP}$ values if we had genotyped additional SNPs (Visscher et al., Ref 30). It is explainable in terms of substantive theory, since for a polygenic trait, inclusion of more SNPs is expected to include more causal polymorphisms into calculating the genetic relationship matrix **A**—either directly or by proxy due to LD. This is supported by a study of several quantitative phenotypes by Yang et al. (2011, *Nature Genetics 43*, 519-525), in which they partitioned σ^2_g among the 22 autosomes and observed positive correlations between each chromosome's length and its own variance component (e.g., $r = 0.83$ for height). Further analyses suggested that these correlations reflected not chromosomal length *per se*, but the number of intragenic SNPs genotyped on each chromosome.

With MZ twins included in analysis, *GCTA* produced $\hat{h}^2_{SNP}$ estimates of 0.77, right around the residual MZ-twin correlation from *RFGLS* (Table S2, above), 0.80. A previous study of FSIQ in a sample of MTFS twins and SIBS siblings (Kirkpatrick et al., 2009, *Behavior Genetics 39*, 406-416) estimated standardized biometric variance components of $a^2 = 0.61$ and $c^2 = 0.18$, which together sum to 0.79. It would appear that Yang et al. were exactly right in their warning that including close relatives in a *GCTA* analysis confounds the shared environment with aggregate SNP effects.

Figure A1. *GCTA* $\hat{h}^2_{SNP}$ as calculated at different genetic-relatedness ceilings.

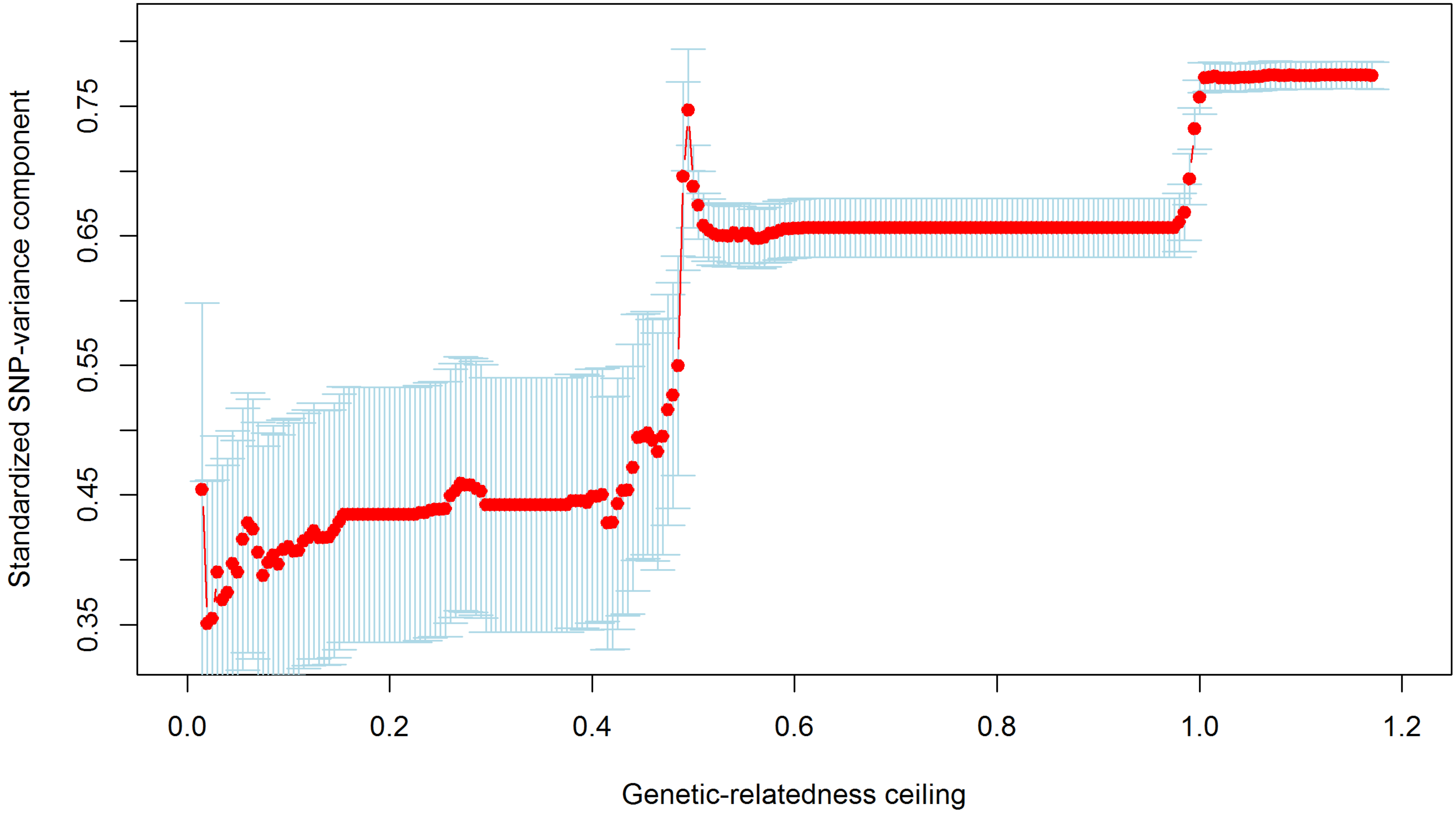

Error bars are ±1 standard error. Genetic-relatedness ceiling is the maximum degree of genetic relationship allowed among participants entered into analysis.

Figure A2. *GCTA* sample size as function of genetic-relatedness ceiling.

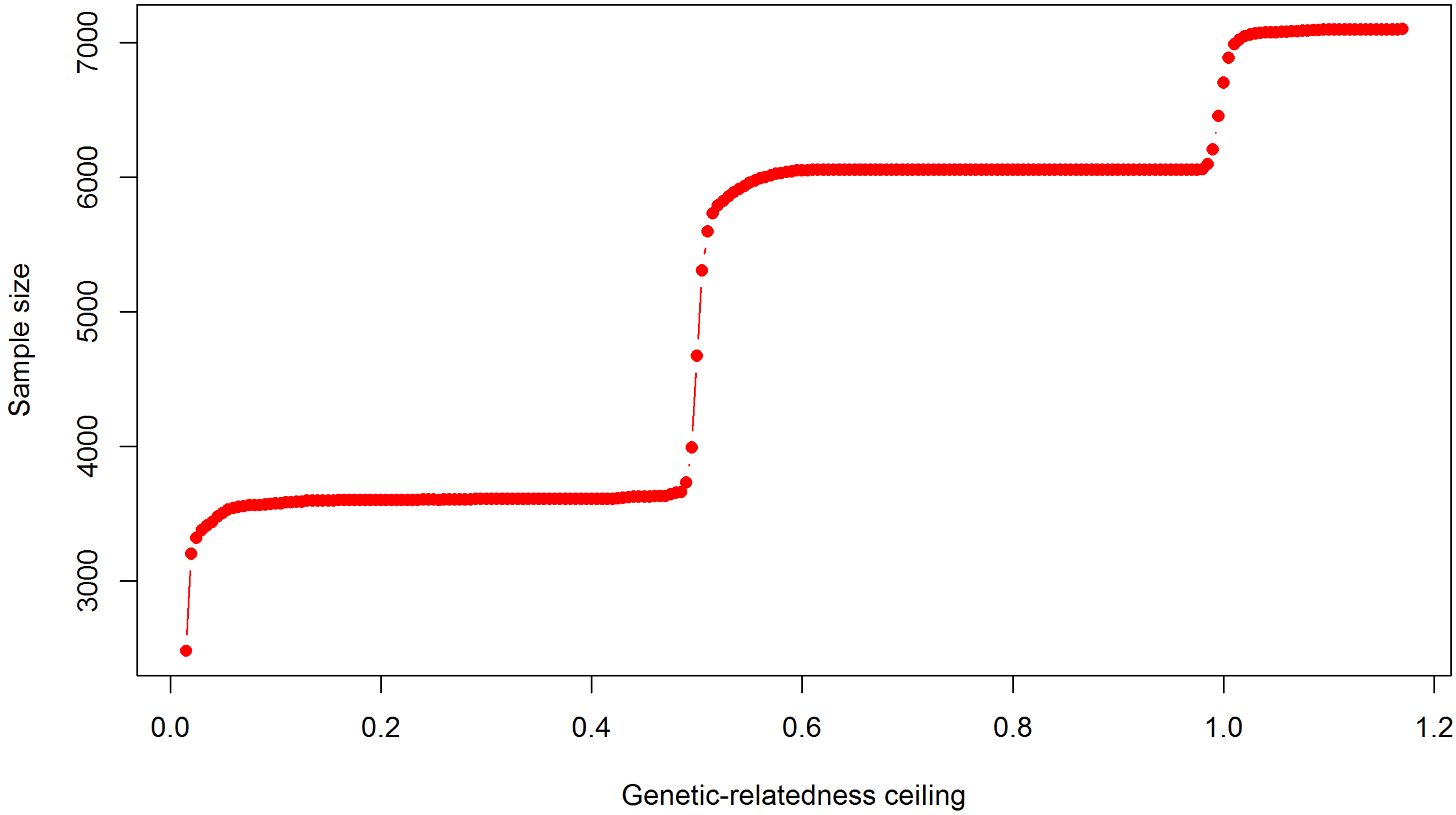

Genetic-relatedness ceiling is the maximum degree of genetic relationship allowed among participants entered into analysis.

Figure A3. *GCTA* $\hat{h}^2_{SNP}$ as calculated at different genetic-relatedness ceilings, graphed as a function of sample size.

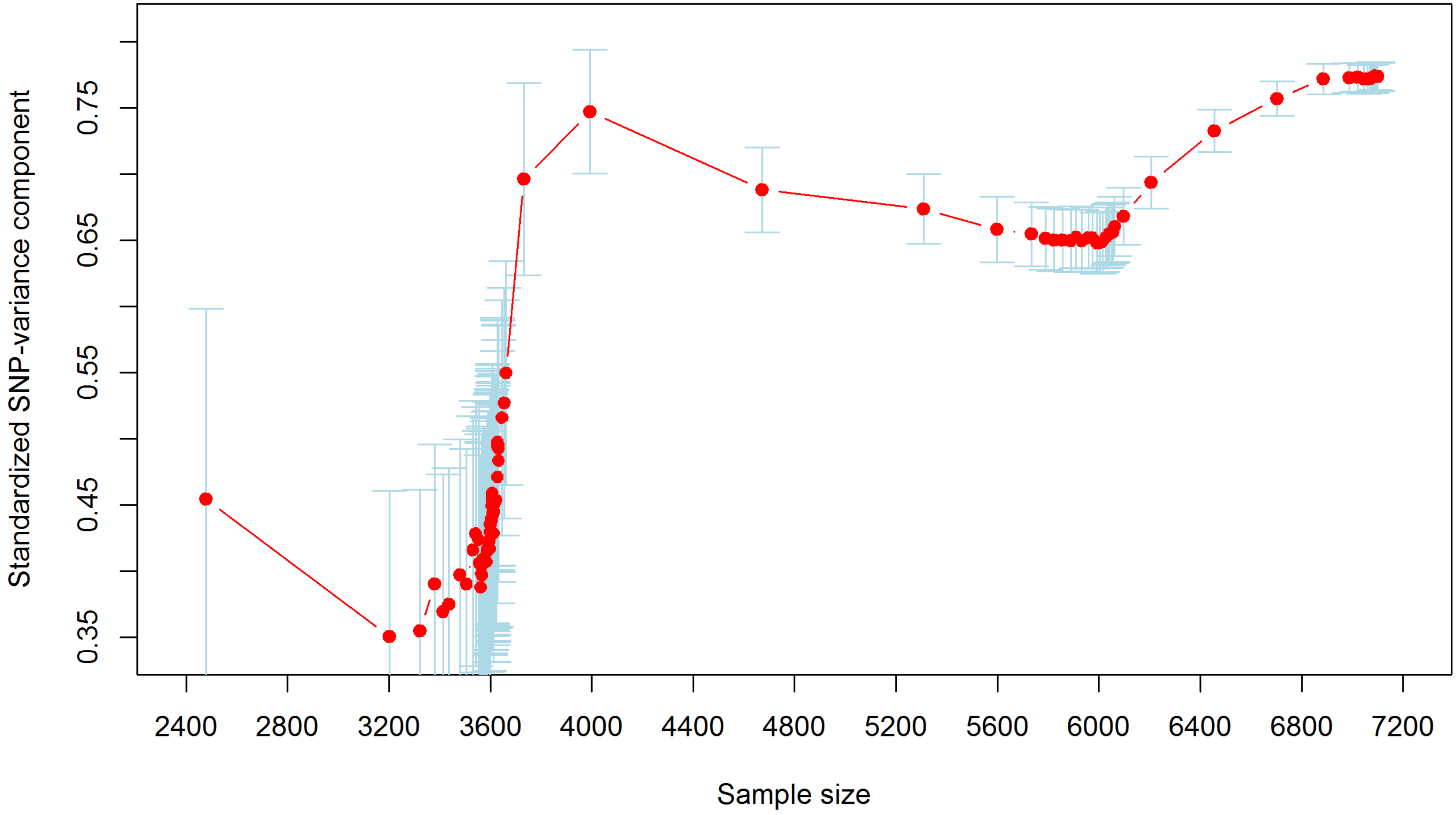

Error bars are ±1 standard error. Sample size was not manipulated directly, but was instead a consequence of genetic-relatedness ceiling. Genetic-relatedness ceiling is the maximum degree of genetic relationship allowed among participants entered into analysis.
